# Distinct proliferative and neuronal programmes of chromatin binding and gene activation by ASCL1 are cell cycle stage-specific

**DOI:** 10.1242/dev.204816

**Published:** 2025-06-25

**Authors:** William F. Beckman, Lydia M. Parkinson, Lewis Chaytor, Anna Philpott

**Affiliations:** ^1^ Wellcome-MRC Cambridge Stem Cell Institute, Jeffrey Cheah Biomedical Centre, Cambridge Biomedical Campus, Cambridge CB2 0AW, UK; ^2^ Department of Oncology, University of Cambridge, Cambridge CB2 0AH, UK

**Keywords:** ASCL1, Neuroblastoma, Differentiation, Cell cycle

## Abstract

ASCL1 is a potent proneural factor with paradoxical functions during development, promoting both progenitor pool expansion and neuronal differentiation. How a single factor executes and switches between these potentially opposing functions remains to be understood. Using human neuroblastoma cells as a model system, we show that ASCL1 exhibits cell cycle phase-dependent chromatin binding patterns. In cycling cells, S/G2/M phase-enriched binding occurs at promoters of transcribed pro-mitotic genes, while G1 phase-enriched binding of ASCL1 is associated with the priming of pro-neuronal enhancer loci. Prolonged G1 arrest is further required to activate these ASCL1-bound and primed neuronal enhancers to drive neuronal differentiation. Thus, we reveal that the same transcription factor can control distinct transcriptional programmes at different cell cycle stages, and demonstrate how lengthening of G1 allows engagement of a differentiation programme by turning unproductive factor binding into productive interactions.

## Teaser

**Highlighted Article:** ASCL1 binds to distinct targets in different cell cycle phases, controlling the switch from division to differentiation in neuroblastic cells in response to G1 lengthening.

## INTRODUCTION

Achaete-Scute homolog 1 (ASCL1) is a potent driver of neurogenesis in both the central and peripheral nervous system (CNS/PNS) and has emerged as a key player in the balance between neuronal progenitor cell differentiation and self-renewal. Strict spatiotemporal orchestration of these processes is essential for the development of the mammalian nervous system to ensure that a sufficient number of neurons are generated at appropriate stages, without progenitor pool depletion (Belmonte-Mateos and Pujades, 2022). ASCL1 is necessary and sufficient to activate neuronal differentiation and specify subtype identity (Cai et al., 2000; Farah et al., 2000; Bertrand et al., 2002; Nakada et al., 2004; Wilkinson et al., 2013; Raposo et al., 2015). Perturbation of the physiological activity of ASCL1 results in atrophy or delayed development of CNS and PNS components (Sudarov et al., 2011; Memic et al., 2016). In the PNS, ASCL1 is transiently expressed in the precursors of enteric and sympathetic neurons, including noradrenergic neuroblasts, and is subsequently downregulated concomitantly with neuronal differentiation (Lo et al., 1991; Pattyn et al., 2004; Wylie et al., 2015; Parkinson et al., 2022). In the CNS, ASCL1 controls fate specification of neuronal and glial lineages, where knockout impairs the normal development of multiple brain regions (Pattyn et al., 2006; Kim et al., 2008; Castro et al., 2011; Azzarelli et al., 2022). Here, ASCL1 is also transiently expressed in mitotic neuronal precursors, with the onset of this expression coinciding with the transition of quiescent stem cells to immature progenitors (Kim et al., 2007), and with subsequent downregulation of ASCL1 coinciding with terminal differentiation and cell cycle exit (Kim et al., 2008). *Ascl1*-null mice show a reduction in the number of cycling intermediate neural progenitor cells in the subventricular zone of the ventral telencephalon, stemming from reduced expression of ASCL1 targets encoding components of the E2F1 and FoxM1 pathways (Castro et al., 2011). ASCL1 was also shown to bind and activate many genes involved in cell cycle progression, such as *E2f1*, *Cdk1* and *Cdc25b* (Castro et al., 2011).

Neuronal differentiation is tightly and inversely linked to cell cycle progression, to the extent that many of the archetypal cell cycle regulators also have non-canonical roles in neuronal specification (Zhu and Skoultchi, 2001; Hardwick and Philpott, 2014). Repurposing of these cell cycle regulators alters cell cycle structure; indeed, lengthening of G1 phase has repeatedly been shown to promote differentiation in somatic stem cells (Lange and Calegari, 2010; Liu et al., 2019; Swadi et al., 2019; Ferguson et al., 2023), and rapid transition through G1 phase is a hallmark of the pluripotent state (Coronado et al., 2013). Mechanistically, the effect of G1 length on specification derives from the capacity of lineage-defining pioneer factors to prime cell type specific enhancers in G1 (Singh et al., 2013), but requires a lengthening of G1 phase to fully commission them (Balsalobre and Drouin, 2022). This primed enhancer state is associated with low levels of H3K4me1 and chromatin accessibility (Balsalobre and Drouin, 2022), where activation leads to deposition of H3K4me1, H3K27ac and increased accessibility, in part due to recruitment of SWI/SNF by enhancer RNAs (eRNAs) which in turn recruits MLL3/4 (KMT2C/D), p300/CBP (EP300/MYCBP) and the mediator complex (Saha et al., 2024), initiating determinate cell type trajectories.

We have previously shown that ASCL1 is capable of performing both context-dependent proliferative and differentiation functions in neuroblastoma, a paediatric cancer originating from immature sympathoadrenal precursors of the PNS (Matthay et al., 2016; Jansky et al., 2021), where ASCL1 is normally a member of the adrenergic subtype core regulatory gene circuit driving growth (Boeva et al., 2017; van Groningen et al., 2017; Wang et al., 2019). In neuroblastoma cells, knockout of ASCL1 reduces *in vitro* proliferation rate (Parkinson et al., 2022), while overexpression drives potent cell cycle exit and neuronal differentiation (Ali et al., 2020; Woods et al., 2022). This ability to drive differentiation is further enhanced when ASCL1 multi-site phosphorylation is prevented (Ali et al., 2014, 2020; Woods et al., 2022), showing that post-translational modifications of ASCL1 in response to cell cycle-dependent CDK2 may regulate its activity, and indicating the possibility that ASCL1 function may be differentially controlled at different stages of the cell cycle. Thus, neuroblastoma cells provide a convenient model system to test whether the potentially conflicting functions of ASCL1 in proliferation and differentiation could be explained by differential activities of this transcription factor at different phases of the cell cycle. To investigate potential cell cycle stage-dependent functions of ASCL1, we have performed ChIP-seq and RNA-seq to detect ASCL1 binding and gene activation in neuroblastoma cells at different cell cycle stages. In SG2M, ASCL1 binding sites are enriched at promoters of cell cycle-associated genes, which supports their expression. In contrast, ASCL1 binding sites enriched in G1 show more association with enhancers of neuronal function genes. However, in cycling cells these G1 ASCL1-bound genes are poorly expressed and remain relatively inaccessible, showing gene target activation only after cells are arrested in G1.

## RESULTS

### ASCL1 binds to both pro-proliferative and pro-neuronal loci in asynchronous cycling cells

**Fig. 1.**
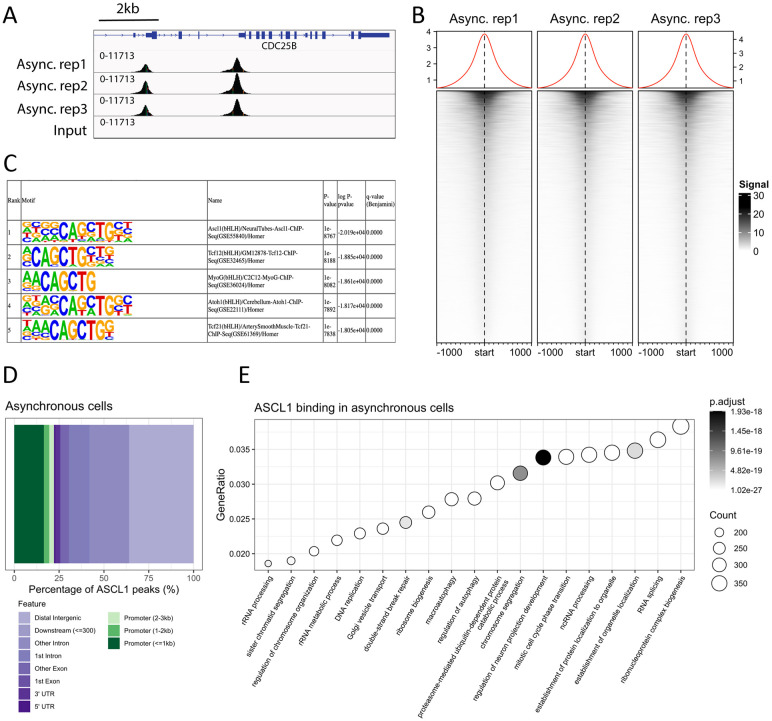
**ASCL1 binds both proliferative genes and neuronal genes in asynchronous cell populations.** (A) ASCL1 ChIP-seq tracks of biological replicates in asynchronous SK-N-BE(2)-C cells, plus input control. (B) ASCL1 ChIP-seq heatmaps for three asynchronous replicates over the consensus peak set. (C) Motif analysis of the consensus ASCL1 peak set (present in two out of three replicates). (D) ASCL1 ChIP-seq consensus peak set annotation in asynchronous cells coloured by feature. (E) GO analysis of ASCL1 ChIP-seq peaks in asynchronous cells annotated to their nearest expressed gene TSS within 50 kb.

We have previously shown that ASCL1 overexpression in neuroblastoma cell lines results in its widespread binding near loci associated with neuronal structures and functions, leading to subsequent neuronal differentiation. However, ASCL1 is expressed at readily detectable levels in many rapidly cycling adrenergic-type neuroblastoma cell lines, where it supports their proliferation (Parkinson et al., 2022). We currently have limited understanding of endogenous ASCL1 binding and how it supports gene expression in proliferating neuroblastoma cells (Wang et al., 2023). To investigate this, we performed ChIP-seq for ASCL1 on cycling SK-N-BE(2)-C cells, a representative MYCN-amplified neuroblastoma cell line. Approximately 100,000 high confidence ASCL1 binding sites were found to be present in two out of three replicates (Fig. 1A,B), and the majority of these sites harboured canonical ASCL1 E-box motifs (Fig. 1C). Of these ∼100,000 peaks, 25,179 were also identified as ASCL1 binding sites in human cortical neurons of the CNS (Păun et al., 2023) (Fig. S1A). Consistent with the known role of ASCL1 in binding distal gene regulatory elements (Raposo et al., 2015), we found that only ∼25% of these high confidence binding sites were within 3 kb of transcription start sites (TSSs), with most binding sites being intergenic (Fig. 1D). To identify potential direct regulatory targets of ASCL1, we limited gene annotation to the nearest expressed gene within 50 kb of each peak (Fig. S1B), leaving 50,488 peaks annotated to 11,950 genes. Gene Ontology (GO) analysis of these bound genes in asynchronous cycling cells at all cell cycle stages revealed that ASCL1 binding is associated most prominently with core processes such as ribonucleoprotein complex biogenesis and RNA splicing, proliferative processes such as chromosome segregation and DNA replication, and pro-neuronal processes such as the regulation of neuron projection development (Fig. 1E). We performed ASCL1 ChIP-seq in another MYCN-amplified neuroblastoma cell line (IMR-32) and a non MYCN-amplified cell line (SH-SY5Y), showing that ASCL1 binding at loci associated with both proliferative and neuronal genes is a general feature of ASCL1 functionality in neuroblastoma (Fig. S1C).

**Fig. 2.**
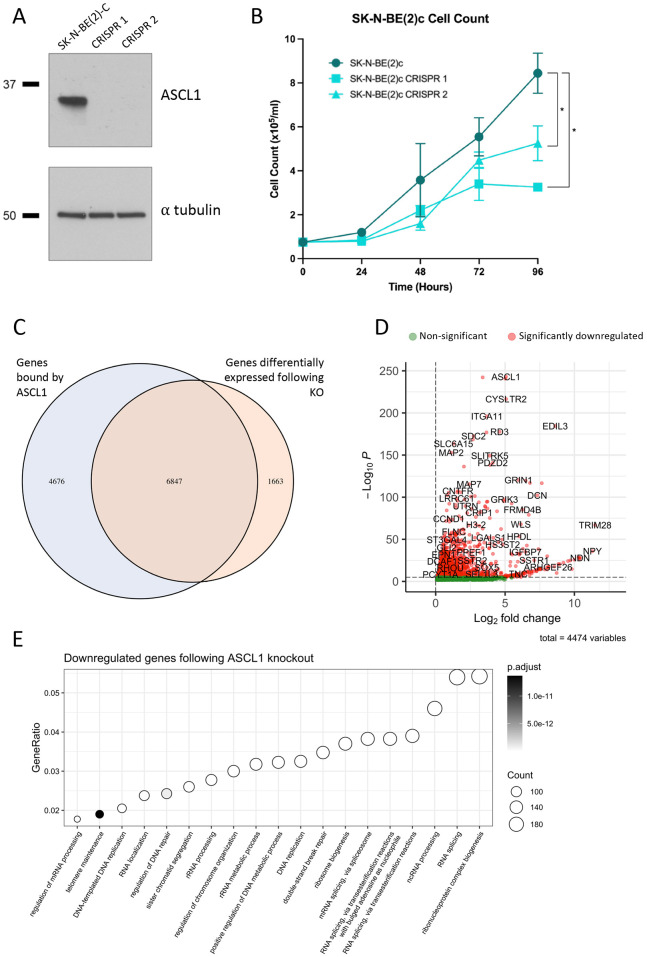
**ASCL1 drives cell cycle progression in proliferating neuroblastoma cells.** (A) ASCL1 western blot in parental SK-N-BE(2)-C cells and two ASCL1 CRISPR knockout clones. (B) Cell proliferation assay for SK-N-BE(2)-C parental cells and both ASCL1 CRISPR knockout clones. Mean values are shown (*n*=3) and s.e.m. (unpaired two-tailed *t*-test; *P<0.05). (C) Intersection between genes bound by ASCL1 and genes significantly differentially expressed following ASCL1 CRISPR knockout. (D) RNA-seq results comparing ASCL1 wild-type SK-N-BE(2)-C and the ASCL1 knockout clone. Only genes showing a fold change of >0 are shown, with significant genes in red and non-significant genes in green. (E) GO analysis of downregulated genes following ASCL1 CRISPR knockout.

To ascertain whether ASCL1 binding at these loci regulated gene expression in cycling neuroblastoma cells, we generated two ASCL1 knockout SK-N-BE(2)-C clones and validated ASCL1 protein absence by western blot (Fig. 2A). While there were no obvious morphological changes in these cells (Fig. S1D,E), knockout of ASCL1 significantly reduced the growth rate when compared to wild-type cells (Fig. 2B), indicating that ASCL1 indeed plays a role in promoting cell cycle progression, as has been seen in other neuroblastoma cell lines (Parkinson et al., 2022). We next performed RNA-seq on the parental cell line and one of the ASCL1 knockout clones (CRISPR 1), identifying 8010 genes that were significantly differentially expressed following knockout, of which the majority (6847) are associated with ASCL1 binding sites in asynchronous wild-type cells (Fig. 2C). Genes associated with the strongest ASCL1 binding sites were generally downregulated following ASCL1 knockout (Fig. S2). GO analysis of all the 4474 genes downregulated after ASCL1 knockout (Fig. 2D) uncovered similar terms to those identified from the ASCL1 ChIP-seq peak set including RNA and ribosomal terms, strongly supporting a role for ASCL1 in regulating these biological processes by directly binding constituent genes and their regulatory elements (Fig. 2E). Interestingly, none of the top 50 terms identified following GO analysis of downregulated genes related to neuronal processes or functions, indicating that ASCL1 binding is not required for expression of neuronal genes in asynchronous freely cycling cells. However, many pro-proliferative and mitotic terms, including DNA replication and sister chromatid separation, were identified in the GO analysis of downregulated genes, suggesting that ASCL1 actively drives cell cycle progression in proliferating neuroblastoma cells.

### ASCL1 binding profiles in transiently synchronised cells recapitulates binding dynamics in cycling cells

**Fig. 3.**
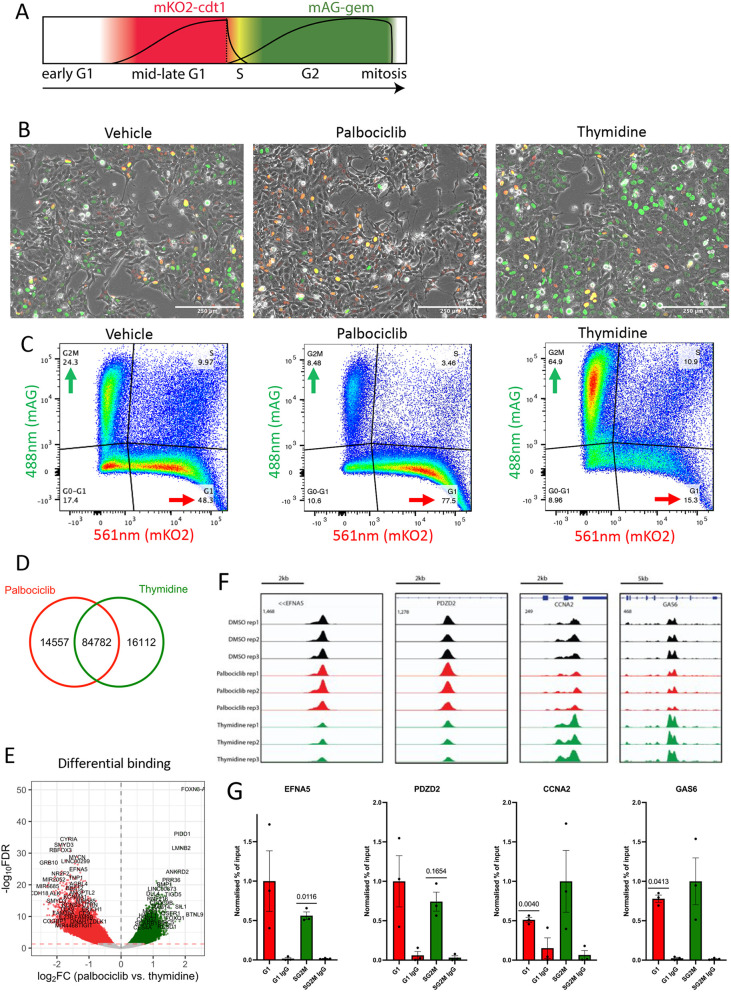
**Cell cycle synchronisation mirrors ASCL1 binding dynamics in cycling cells.** (A) Schematic of FUCCI cell cycle reporter system. (B) Fluorescence images of SK-N-BE(2)-C FUCCI reporter cells following DMSO (vehicle, left), palbociclib (centre) and thymidine (right) treatments. Scale bars: 250 µm. (C) Flow cytometry analysis of SK-N-BE(2)-C FUCCI reporter cells after DMSO (vehicle, left), palbociclib (centre) and thymidine (right) treatments. (D) Venn diagram depicting ASCL1 ChIP-seq consensus peaks (present in two out of three replicates) overlap between palbociclib- and thymidine-treated cells. (E) Volcano plot of ASCL1 ChIP-seq peaks following DiffBind analysis. Red dotted line represents −log_10_(0.05) *P*-value. Green points represent thymidine-enriched loci and red points represent palbociclib-enriched loci. (F) Replicate ASCL1 ChIP-seq tracks for DMSO (black), palbociclib-treated (red) and thymidine-treated (green) cells around two palbociclib (left) and two thymidine (right) enriched sites. (G) ASCL1 ChIP-qPCR for the four sites shown in E on FACS sorted G1 or SG2M cells (see Materials and Methods). Error bars show s.e.m. (*n*=3, one sample two tailed *t*-test).

Given that SK-N-BE(2)-C cells represent immature neuronal precursors and ASCL1 has been extensively investigated as a neurogenic factor (Rao et al., 2021; Vainorius et al., 2023; Wohlschlegel et al., 2023), we sought to elucidate how ASCL1 can bind to and promote the expression of pro-proliferative genes while simultaneously binding neuronal loci without engaging a neuronal differentiation programme. We hypothesised that the binding profile determined from asynchronously cycling cells may represent two or more distinct ASCL1 binding profiles, each being derived from cells at different phases of the cell cycle. To test this, we generated a SK-N-BE(2)-C cell line stably harbouring the FUCCI cell cycle dual reporter system, allowing us to assign individual cells to distinct cell cycle phases, and monitor cell cycle progression (Sakaue-Sawano et al., 2008). The FUCCI system consists of a green fluorophore (mAG) tagged with a geminin-derived degron, and a red fluorophore (mKO2) tagged with a Cdt1-derived degron. Hence cells will acquire an overall red fluorescence when they are in (mid-late) G1 phase, and a green fluorescence during G2 and M phases. Cells in early G1 phase will accumulate neither the red nor the green fluorophore, whereas cells cycling through S phase will exhibit both red and green fluorescence and appear yellow, and stalling cells in S phase will result in overall green fluorescence (Fig. 3A) (McGarry and Kirschner, 1998; Takeda et al, 2005).

We used the CDK4/6 inhibitor, palbociclib, to stall the cells in G1 phase resulting in an increase in mKO2-positive cells, and thymidine to stall the cells in S phase resulting in an increase in mAG-positive cells (Fig. 3B,C). DNA staining and karyotyping using Hoechst 33342 revealed that palbociclib treatment reduced the fraction of cells with replicated DNA (4c) characteristic of G2M phase cells, while thymidine treatment resulted in an accumulation of cells with a DNA content consistent with those expected in S phase cells, where chromosomes have been partially replicated (Fig. S3A). Removal of both drugs resulted in detectable re-entry into the cell cycle within 3 h (Fig. S3B-D), demonstrating cell viability and drug reversibility (Ligasová and Koberna, 2021; Crozier et al., 2022).

After stalling cells in G1 or S phase, inhibitors were washed out to mitigate any direct effects of the drugs on transcription factor binding, and ChIP-seq for ASCL1 was performed on the enriched populations fixed 45 min post-drug removal. The majority of ASCL1 peaks were common between the consensus peak sets of palbociclib- and thymidine-stalled cells (Fig. 3D), but DiffBind analysis identified 47,294 differentially bound sites between the two conditions (Fig. 3E). Four of these sites (EFNA5 and PDZD2, enriched in palbociclib samples, and CCNA2 and GAS6, enriched in thymidine samples) were selected for validation by ChIP-qPCR in populations of cells that were fluorescence-activated cell sorting (FACS) sorted according to cell cycle stage (Fig. 3F), based on expression of the FUCCI reporters. Three out of four target sites showed significant cell cycle stage-specific binding that recapitulated the differential pattern seen after palbociclib or thymidine-induced stalling, while the fourth site showed a trend consistent with this differential binding, but did not meet statistical significance (Fig. 3G). We therefore concluded that the cell cycle synchronisation approach using drugs reveals cell cycle-dependent differences in ASCL1 binding that are also seen at different cell cycle stages in asynchronously cycling cells.

### ASCL1 preferentially binds to neuronal loci during G1 phase, and pro-proliferative loci during SG2M phase of the cell cycle

**Fig. 4.**
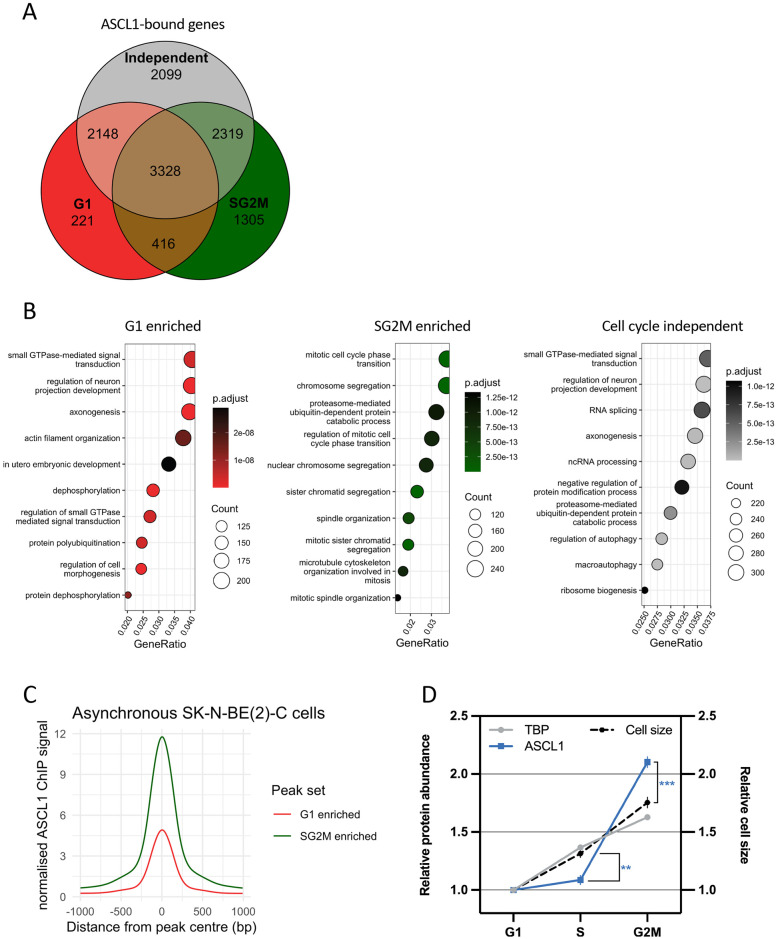
**ASCL1 binds neuronal genes during G1 phase and proliferative genes during SG2M phase of the cell cycle.** (A) Venn diagram of genes with G1 enriched (red), SG2M enriched (green) or cell cycle independent (grey) ASCL1 ChIP-seq associated peaks. (B) GO analysis of G1 enriched (red), SG2M enriched (green) and cell cycle independent (grey) ASCL1 ChIP-seq peaks. Only peaks within 50 kb of an expressed gene were used. (C) Normalised ASCL1 ChIP-seq signal in asynchronous cells for sites showing G1 enriched binding (red) and SG2M enriched binding (green) of ASCL1. (D) ASCL1 protein levels (blue) and TBP protein levels (grey) in the various phases of the cell cycle (as determined by Hoechst 33342 staining and flow cytometry). Relative changes in protein levels compared to relative changes in cell size (black dotted line). Mean values are shown (*n*=3) and s.e.m. (unpaired two-tailed *t*-test; **P<0.002, ***P<0.0004).

We next sought to determine the potential significance of cell cycle stage-specific binding of ASCL1 to the genome. Of the 47,294 sites identified as differentially bound by ASCL1 in G1 versus SG2M cells, 32,834 sites showed higher levels of ASCL1 binding during G1 phase, and 14,460 showed higher levels of ASCL1 binding during SG2M. We then defined all peaks with a false discovery rate (FDR) of over 0.05 as showing cell cycle-independent binding between the two cell cycle stages. All the peaks in these three subsets (G1 enriched, SG2M enriched and cell cycle-independent) were assigned to the nearest expressed gene TSS using a 50 kb distance cut-off. These three gene sets were found to be non-mutually exclusive, as many genes associated with G1 or SG2M enriched peaks also had cell cycle-independent peaks (Fig. 4A). GO analysis of associated genes revealed that that G1 enriched peaks were associated with genes involved in axonogenesis and neuronal differentiation processes, including small GTPase-mediated signal transduction and actin filament organisation, which are crucial for neuronal morphogenesis and polarity (Gentile et al., 2022; Gonzalez-Billault et al., 2012). In contrast, SG2M enriched peaks were overwhelmingly associated with pro-proliferative and mitotic genes rather than neuronal genes. Cell cycle-independent peaks were associated with more varied functions including metabolic and ribosomal, but also some neuronal processes (Fig. 4B). The same trend was observed when assigning peaks to their nearest TSS irrespective of distance (Fig. S4A): axonal terms did appear in the SG2M ontology analysis alongside pro-proliferative terms in this less stringent analysis.

Despite asynchronous cells comprising ∼65% G0/G1 cells versus 35% SG2M cells, we found that in asynchronous ASCL1 ChIP-seq data, peaks enriched for ASCL1 binding in G1 showed generally lower levels of ASCL1 binding compared to those enriched in SG2M (Fig. 4C), which was replicated in two other neuroblastoma cell lines (Fig. S4B). We hypothesised that the lower levels of ASCL1 binding in asynchronous cells for G1 enriched sites relative to SG2M sites was a result of lower ASCL1 protein levels in G1 phase compared to SG2M. To test this, we performed ASCL1 antibody-based staining and Hoechst 33342-based cell cycle analysis in SK-N-BE(2)-C cells, followed by flow cytometry. This enabled us to individually quantify ASCL1 protein levels in specific cell cycle subpopulations. The relative cell size changes across the cell cycle, so to account for this we compared the relative changes in ASCL1 protein levels with the relative changes in cell size. This revealed that ASCL1 protein levels in G2M were significantly higher than expected due to changes in cell size (Fig. 4D) and as compared to G1 ASCL1 levels. In contrast, when we performed the same analysis for the housekeeping gene, *TBP*, we observed more consistent protein levels that scaled proportionately with cell size (Fig. 4D). This reveals a degree of cell cycle-dependent regulation of ASCL1 protein levels, which may account for differences in overall binding between the two phases, indicating that reduced binding in G1 may be due to a lower concentration of ASCL1 protein in this cell cycle phase compared to other phases.

**Fig. 5.**
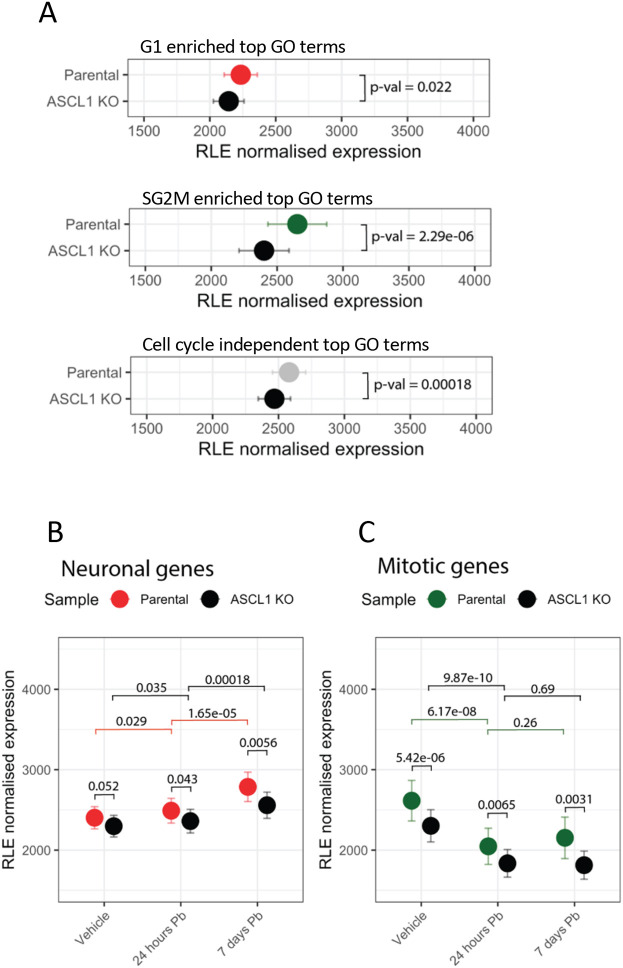
**ASCL1-dependent switch from mitotic to neuronal specific gene expression in G1 arrested neuroblastoma cells.** (A) RNA-seq expression of genes derived from the top ten GO terms from Fig. 4B in parental (coloured) or ASCL1 knockout (black) cells. Error bars represent s.e.m. (merge of four replicates, two tailed *t*-test). (B) RNA-seq expression in parental (red) or ASCL1 knockout (black) cells for genes derived from neuronal GO terms of G1 enriched ASCL1 ChIP-seq peaks following palbociclib treatment. Error bars represent s.e.m. (merge of four replicates, two tailed *t*-test with Bonferroni multiple testing correction; *P*adj=0.0071; see Materials and Methods). (C) RNA-seq expression in parental (green) or ASCL1 knockout (black) cells for genes derived from mitotic GO terms of SG2M enriched ASCL1 ChIP-seq peaks following palbociclib treatment. Error bars represent s.e.m. (merge of four replicates, two-tailed *t*-test with Bonferroni multiple testing correction; *P*adj=0.0071; see Materials and Methods).

To investigate the dependency of these genes on ASCL1 binding for expression, we took genes in the top 10 GO terms associated with ASCL1 binding in G1, SG2M or exhibiting cell cycle-independent binding, and analysed their expression in the ASCL1 knockout clone compared to cycling parental cells expressing endogenous ASCL1. The gene sets associated with cell cycle-independent ASCL1 binding or those with enriched ASCL1 binding during SG2M were significantly downregulated in ASCL1 knockout cells, indicating that they normally rely on endogenous ASCL1 binding for expression. In contrast, genes with enriched ASCL1 binding during G1, including neuronal genes, did not show a significant decrease in expression level after ASCL1 knockout (Fig. 5A). This indicates that ASCL1 is capable of binding to neuronal targets in G1 phase of the cell cycle in neuroblastoma cells but is not supporting their expression under cycling conditions.

### Prolonged G1 arrest converts primed ASCL1 binding at neuronal genes into productive binding

Differentiation is associated with an elongated G1 phase (Lange and Calegari, 2010), and we have previously shown that G1 arrest can trigger a re-engagement of the differentiation process in neuroblastoma cells (Ferguson et al., 2023). Hence, we hypothesised that in the G1 phase of cycling cells, ASCL1 may be marking neuronal genes for potential expression, but the short duration of G1 phase in these neuroblastic cells may be insufficient to allow ASCL1 binding to activate neuronal gene expression.

To investigate whether prolonged arrest in the G1 phase is required to enable ASCL1 to activate expression of these ASCL1-bound, G1-enriched neuronal genes, we performed RNA-seq after 24 h and 7 days of palbociclib treatment, comparing gene expression in ASCL1 wild-type and knockout cells. We have previously extensively characterised the effects of palbociclib treatment of SK-N-BE(2)-C cells, where it results in a strong reduction in cell cycle activity and acquisition of neuronal traits, such as neurite extension (Ferguson et al., 2023). Consistent with these previous findings, we observe a strong downregulation of stemness markers *SOX2*, *MYCN* and *HES1*, and a strong upregulation of neuronal genes *DCX* and *MAP2* following 7 days of palbociclib treatment (Fig. S5A), which may represent the increased fraction of neurons versus progenitor cells in the culture. Focussing on genes associated with neuronal functions that are preferentially bound by ASCL1 during G1 phase compared to SG2M, we found that their expression was significantly increased after 7 days of palbociclib treatment (Fig. 5B). Crucially, expression of this gene set was not significantly different in ASCL1 knockout cells growing asynchronously (Fig. 5B, vehicle) or after 24 h of palbociclib treatment. However, cells showed significantly more upregulation of neuronal genes after 7 days of palbociclib treatment when ASCL1 is present, indicating that ASCL1 cannot drive expression of these neuronal genes in neuroblasts unless G1 arrest is prolonged. We also looked at the pro-mitotic genes preferentially bound by ASCL1 during SG2M phase and found that, in contrast to neuronal genes, their expression was ASCL1-dependent in asynchronously cycling cells (Fig. 5C, vehicle). As expected, their expression was significantly reduced by 24 h and remained low after 7 days of palbociclib treatment (Fig. 5C), reflecting the rapid exit of cells from the cell cycle following CDK inhibition. Our data reveal that ASCL1 binds neuronal genes specifically in G1 phase, but it does not drive expression of these genes in cycling neuroblasts, instead requiring prolonged G1 arrest for neuronal gene activation. To rule out any confounding effects from other proneural transcription factors, we quantified their expression levels in ASCL1 wild-type and ASCL1 knockout cells, before and after palbociclib treatment (Fig. S5B), and found that their expression remained very low or undetectable in both cell lines throughout the palbociclib timecourse.

### ASCL1 preferentially interacts with inaccessible distal regulatory elements during G1 phase, and accessible promoter elements during SG2M phase of cycling cells

**Fig. 6.**
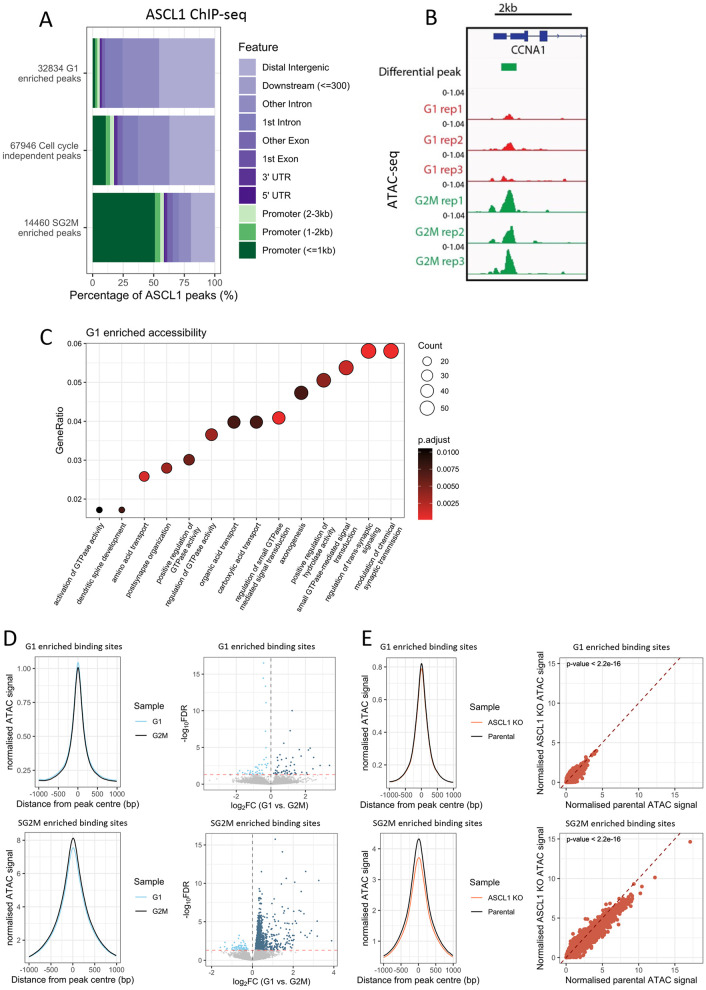
**ASCL1 preferentially binds closed enhancers during G1 phase and open promoters during SG2M phase of the cell cycle.** (A) ASCL1 ChIP-seq peak annotation for G1 enriched (top), cell cycle independent (middle) and SG2M enriched peaks (bottom). (B) Normalised ATAC-seq tracks for G1 (red) and G2M (green) replicates around the *CCNA1* promoter. (C) GO analysis of G1 enriched accessible regions following annotation to their nearest TSS within 50 kb. (D) Normalised ATAC-seq signal for G1 (blue) and G2M (black) sorted cells at ASCL1 bound, G1 enriched (top panels) and SG2M enriched (bottom panels) peaks. Right panels show volcano plots following DiffBind analysis at these sites. Dotted red line represents significance threshold. (E) Normalised ATAC-seq signal for parental SK-N-BE(2)-C (orange) and ASCL1 knockout (black) asynchronous cells at ASCL1 bound, G1 enriched (top panels) and SG2M enriched (bottom panels) peaks. Right panels show average ATAC-seq signal in wild-type or ASCL1 KO cells for each differential ASCL1 ChIP-seq peak (two-tailed paired *t*-test). Dotted lines represent *x*=*y*.

Given the different nature of the genes targeted by ASCL1 in G1 and SG2M and the differential reliance on endogenous ASCL1 in cycling cells to support their expression, we wanted to investigate the genomic context of ASCL1 enriched binding sites between G1 and SG2M. We returned to the previously identified cell cycle-independent, G1-enriched and SG2M-enriched ASCL1 ChIP-seq peaks (Fig. 4B) and investigated peak locations in relation to known gene positions and TSSs. We found that 15% of cell cycle-independent peaks were found within 3 kb of a known TSS, while 85% were located in exonic and intergenic regions (Fig. 6A). Surprisingly, G1 enriched peaks were almost entirely annotated to non-promoter regions, with only ∼5% of binding sites occurring within 3 kb of a known TSS, whereas the composition of SG2M peaks differed substantially, with ∼60% of SG2M enriched ASCL1 binding occurring around a known TSS (Fig. 6A).

If ASCL1 can bind to distal regulatory elements during G1 phase but cannot activate associated genes in cycling cells, one might predict that accessibility levels at these sites would be low under these conditions, especially when compared to promoter regions of ASCL1-dependent genes bound preferentially by ASCL1 during SG2M. To test this, we FACS sorted cycling cells into enriched G1 phase or G2M phase populations based on DNA content and performed ATAC-seq (Fig. 6B) to identify regions of the genome that show differential accessibility at different cell cycle phases. DiffBind analysis using an FDR corrected *P*-value threshold of 0.1 identified 1996 G1 phase and 1560 G2M phase enriched accessible sites (Fig. S6A). Echoing our findings for differential ASCL1 binding sites at different cell cycle stages, we saw that sites that were more accessible during G1 phase were more likely to be distal from TSSs, whereas sites that were more accessible during G2M were more likely to be within 3 kb of a TSS (Fig. S6A). GO analysis revealed G1 enriched accessible sites to be associated with genes involved in GTPase signalling and neuronal processes (Fig. 6C), whereas G2M enriched accessible regions were not associated with any specific GO category.

To ascertain whether ASCL1 binding affects differential accessibility at these sites at different cell cycle stages, we took all sites enriched for ASCL1 binding during G1 and all sites enriched for ASCL1 binding during SG2M and compared their accessibility profiles with ATAC-seq profiles from G1 and G2M cells. Chromatin regions at peaks showing higher levels of ASCL1 binding during G1 phase were more accessible during G1 phase than in G2M, while peaks showing higher levels of ASCL1 binding during SG2M phase were more accessible during G2M phase than during G1 phase. This indicates that ASCL1 binding does contribute to some modest differences in accessibility at different cell cycle stages. Nevertheless, on average, G1 ASCL1-bound sites had lower levels of accessibility than SG2M bound sites (Fig. 6D, left panels). To validate a potential functional role of ASCL1 in contributing to accessibility at ASCL1-bound sites, we performed ATAC-seq on asynchronous ASCL1 wild-type or ASCL1 knockout cells. Analysis of the accessibility profiles at ASCL1-bound G1 phase enriched and SG2M phase enriched sites revealed a significant decrease in accessibility at both the G1 sites and SG2M sites in the absence of ASCL1. However, the change in accessibility at G1 sites was modest (Fig. 6E) in agreement with the modest changes seen when comparing G1 and G2M accessibility (Fig. 6D). We selected only accessible regions containing a canonical ASCL1 E-box motif (NNVVCAGCTGBN, taken from HOMER motif analysis in Fig. 1C) and reanalysed the change in accessibility at SG2M sites after ASCL1 knockout (Fig. S6B). The magnitude of the decrease in accessibility was comparable to that seen when using the full set of peaks (Fig. 6D, see discussion).

**Fig. 7.**
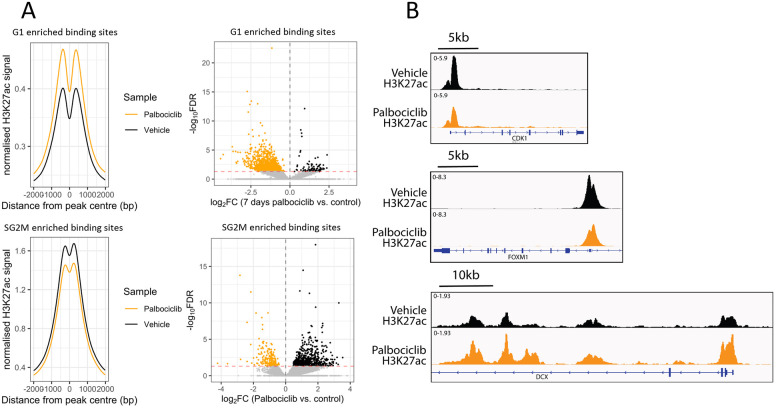
**Prolonged G1 increases H3K27ac mark at ASCL1 bound, G1 enriched sites in SK-N-BE(2)-C cells.** (A) Normalised H3K27ac ChIP-seq signal for control (black) and palbociclib-treated (orange) SK-N-BE(2)-C cells at ASCL1 bound, G1 enriched (top panels) and SG2M enriched (bottom panels) peaks. Right panels show volcano plots following DiffBind analysis of H3K27ac signals at these sites. Dotted red line represents significance threshold. Dotted lines represent *x*=*y*. (B) Merged (*n*=5) H3K27ac ChIP-seq tracks for control (black) and 7 days palbociclib-treated (orange) cells around two cell cycle gene promoters (*CDK1* and *FOXM1*) and one early neuronal gene (*DCX*).

We have shown that ASCL1 binding in G1 is preferentially associated with genes associated with neuronal structures and functions (Fig. 4B). This G1 binding is enriched at intergenic and intronic sites, many of which are likely to be potential enhancers, yet in cycling cells these are generally inaccessible (Fig. 6D) and associated gene transcription is not maintained by ASCL1 (Fig. 5B). Given that extended G1 cell cycle arrest can activate ASCL1-dependent expression of these genes, we hypothesised that the active epigenetic mark H3K27ac would accumulate at the sites preferentially bound by ASCL1 during this extended G1 phase, supporting gene activation that does not occur in cycling cells. To test this, we analysed previously published H3K27ac ChIP-seq data in cycling SK-N-BE(2)-C cells (Ferguson et al., 2023) and compared accumulation of H3K27ac at sites with more ASCL1 binding in G1 compared to sites with more ASCL1 binding in SG2M (Fig. 7A). There was an evident bimodal signal around the G1 enriched sites, which further supported their identity as potential enhancers (Liu et al., 2015), while the SG2M sites showed a less prominent bimodal peak, characteristic of active promoter elements (Liu et al., 2015). Next, we compared H3K27ac signal at cell cycle-enriched ASCL1 binding sites after 7 days of palbociclib treatment (Ferguson et al., 2023). G1 enriched ASCL1-binding sites showed significantly higher levels of H3K27ac after 7 days of palbociclib treatment compared to cycling cells and a more prominent bimodal distribution indicating an overall transition from ASCL1-bound inactive enhancers to active enhancers under these conditions (Fig. 7A). Conversely, SG2M enriched ASCL1-bound sites showed a significant reduction in H3K27ac after palbociclib treatment, mirroring the observed decrease in expression of these target genes (Fig. 5C) and likely deactivation of the pro-proliferative circuitry after G1 arrest. To test this hypothesis, we assessed the H3K27ac signal at the promoter elements of two canonical cell cycle genes (CDK1 and FOXM1) before and after palbociclib treatment which indeed revealed a reduction in the mark at these sites following palbociclib treatment (Fig. 7B), while the early neuronal marker, DCX, showed an increase in H3K27ac levels at the promoter and further downstream.

## DISCUSSION

During development, ASCL1 is a crucial yet enigmatic protein with well characterised roles in neuronal terminal differentiation and immature progenitor cell expansion. How one protein can direct these opposing biological processes has until now remained a mystery. In the present study we have addressed the hypothesis that, in freely cycling cells, ASCL1 displays cell cycle phase-dependent activity, revealing that G1 phase activity is associated more with neuronal differentiation, while SG2M activity associates more with progression of the cell cycle (Fig. 4). Sites with preferential ASCL1 binding during G1 phase tend to be distally located enhancers of neuronal genes exhibiting low levels of accessibility (Fig. 6) and weak but bimodal H3K27ac peaks (Fig. 7), indicative of primed enhancers (Balsalobre and Drouin, 2022). Sites with preferential binding during SG2M, however, tend to be promoter sites of pro-mitotic genes, exhibiting high levels of accessibility and more unimodal H3K27ac peaks. In these cycling cells, ASCL1 drives gene expression of the SG2M subset only, while prolonged G1 arrest is required to commission primed enhancers to drive associated neuronal gene expression and trigger neuronal morphological changes (Fig. 5). ASCL1 knockout reduced the level of accessibility at ASCL1 bound and SG2M enriched sites, supporting a role for ASCL1 in maintaining accessibility, and this effect was irrespective of the presence of an underlying ASCL1 binding motif (Fig. 6). This indicates that even accessible regions with degenerate or no ASCL1 motifs show a reduction in accessibility following ASCL1 knockout and may imply that ASCL1 binding at these regions is mediated or enhanced by a cofactor. The fact that accessibility generally remains at ASCL1 bound and SG2M enriched regions after ASCL1 knockout suggests that other pioneer factors may play a role in cell cycle-dependent binding of ASCL1.

How ASCL1 binding is regulated in a cell cycle phase-dependent manner is yet to be elucidated, but we reveal here that relative ASCL1 protein levels are cell cycle stage-dependent (Fig. 4D), and we have previously shown that the phosphorylation of ASCL1 by CDKs is a key determinant of its differentiation potential (Ali et al., 2014, 2020; Azzarelli et al., 2024; Liu et al., 2024), which itself could influence degradation rate, binding affinity or binding partners. CDK2 has previously been shown to phosphorylate and activate the histone methyltransferase KMT2B, leading to H3K4me3 deposition selectively at developmental genes during late G1 phase in pluripotent stem cells (Singh et al., 2015). Co-occurrence of H3K4me3 and H3K27me3 defines developmental bivalent chromatin domains, which are resolved during lineage specification such that associated genes are either activated or permanently silenced (Blanco et al., 2020). The significance of H3K4me3 at enhancer loci is still being debated (Blanco et al., 2020), but one possibility here is that binding of ASCL1 at G1 enhancer sites is directed by G1 phase H3K4me3 deposition, and that prolonged G1 phase permits ASCL1 to resolve these bivalent domains. ASCL1 levels have been shown to fall during neuronal cell differentiation, and we find the same trend following 7 days of palbociclib treatment (Fig. S5B), which may indicate that once G1 arrest occurs, and ASCL1 has triggered differentiation, the ASCL1 levels must fall to prevent aberrant re-activation of the cell cycle machinery.

That ASCL1 promotes pro-mitotic gene expression and cell cycle progression is at odds with our previous studies showing that ASCL1 overexpression drives potent neuronal differentiation in neuroblastoma cells (Woods et al., 2022). We propose that overexpression enables increased binding and aberrant activation of these ASCL1-bound G1 primed enhancers with corresponding target gene activation, while overexpression has little impact on the already activated cell cycle progression genes.

Taken together, our study reveals cell cycle stage-specific activities of ASCL1 and gives enhanced insight into how the length and structure of the cell cycle influences the balance between proliferation and differentiation of neuroblastic cells.

### Study limitations

Due to the required cell numbers for ChIP-seq, we used pharmacological cell cycle synchronisation methods to enrich cells in specific cell cycle stages. We made sure to remove the drugs with two growth media washes 45 min before performing ChIP-seq and validated the results in cycling asynchronous sorted cells, but there is always the possibility that the drugs had secondary effects on the activity of ASCL1. Additionally, we use a single neuroblastoma cell line as a model of ASCL1 activity, but this represents a pathological cellular state with many genetic and epigenetic aberrations. Thus it would be of interest to repeat this experiment in additional neuroblastoma lines and a more physiologically relevant context such as in neural stem cells.

## MATERIALS AND METHODS

### Cell culture

SK-N-BE(2)-C cells were cultured at 5% CO_2_ at 37°C in DMEM/F12 with L-glutamine and 15 mM HEPES, and further supplemented with 10% foetal bovine serum (FBS) and 1% Penicillin-Streptomycin (for details of key resources see Table S2). Cells were tested regularly for mycoplasma infection by extended culture in the absence of Penicillin-Streptomycin followed by polymerase chain reaction (PCR). Cell lines were authenticated by short tandem repeat testing and comparison to the Cellosaurus database.

### Generation of stable cell lines

Plasmids for the transient expression of the FUCCI dual reporter system were kindly gifted by Ludovic Vallier (Berlin Institute of Health, Germany). GFP was removed from the third generation pLenti-CMV-GFP-Hygro transfer plasmid using BamH1-HF and Sal1-HF and FUCCI reporters were amplified from transient expression plasmids for insertion using the Takara Bio In-Fusion cloning kit (see Table S1 for primer sequences). Lentiviral particles for the FUCCI reporters (pLenti-CMV-mAG-geminin, pLenti-CMV-mKO2-cdt1) were then generated in HEK293T cells. Briefly, 6 µg of each plasmid harbouring TAT, GAG and POL, and REV, plus 12 µg of VSV-G and 42 µg of each transfer plasmid were added to a near confluent T175 flask of HEK293T cells in IMDM media. Resulting viral particles were collected, concentrated and quantified, and cells were transduced using a viral titre of 10 MOI in the presence of 8 µg ml^−1^ polybrene. Single double-positive cells were sorted from the transduced population and a stable clone was generated.

### Cell cycle synchronisation and ChIP-seq

Three million SK-N-BE(2)-C FUCCI reporter cells were plated in 10 cm^2^ dishes and were then either treated with DMSO for 24 h, 500 nM palbociclib dissolved in DMSO for 24 h, or double blocked with 4 mM thymidine for 24 h with a 12 h release in between. After cell cycle synchronisation (or vehicle) all cells were washed twice with warm media then fresh media was added for 45 min before cells were trypsinised and collected. Cells were pelleted and fixed in 1 ml of cold solution A (1% formaldehyde; 50 mM HEPES; 100 mM NaCl; 1 mM EDTA; 0.5 mM EGTA) and fixation was stopped after 10 min then quenched with glycine to a final concentration of 125 mM. Cells were washed twice with ice cold PBS supplemented with cOmplete protease inhibitor then pellets were frozen on dry ice and stored at −80°C.

Next, 50 µl of protein G Dynabeads were used per sample and were washed twice in 1 ml of 0.5% bovine serum albumin (BSA)-PBS solution in protein LoBind tubes before being resuspended in 100 µl 0.5% BSA-PBS per sample. Then 4.5 μg per sample of ASCL1 antibody was added. Bead-antibody solutions were then mixed and left to rotate for ∼6 h at 4°C.

ChIP-seq was performed as previously described (Schmidt et al., 2009). Briefly, cells were thawed then lysed in 1 ml of cold lysis buffer 1 (50 mM HEPES-KOH; 140 mM NaCl; 1 mM EDTA; 10% glycerol; 0.5% Igepal CA-630; 0.25% Triton X-100) supplemented with cOmplete protease inhibitor then rotated at 4°C for 10 min before being centrifuged at 2000 ***g*** for 5 min at 4°C. Pelleted nuclei were then resuspended in 1 ml of cold lysis buffer 2 (10 mM Tris-HCl; 200 mM NaCl; 1 mM EDTA; 0.5 mM EGTA) supplemented with cOmplete protease inhibitor then rotated at 4°C for 10 min before again centrifuging at 2000 ***g*** for 5 min at 4°C. Pelleted nuclei were then resuspended and lysed in 300 µl of cold lysis buffer 3 (10 mM Tris-HCl; 100 mM NaCl; 1 mM EDTA; 0.5 mM EGTA; 0.1% Na-Deoxycholate; 0.5% N-lauroylsarcosine) supplemented with cOmplete protease inhibitor before being sonicated for six cycles in a Bioruptor 300 sonicator. We added 30 μl of 10% Triton X-100 to the lysate, and histone H2B and total mRNA added to final concentrations of 20 μg ml^−1^ and 1 μg ml^−1^, respectively (acting as carrier). Lysate was centrifuged at 21,000 ***g*** for 10 min then supernatant was divided into 30 µl for input, and 300 µl for ASCL1 immunoprecipitation reaction. DNA LoBind tubes were used from this point on in the protocol. Bead-antibody mixes were removed from the cold room and washed three times with ice cold 0.5% BSA-PBS then resuspended in 100 µl of 0.5% BSA-PBS per sample. Then 100 µl of bead-antibody solution was added to the 300 µl of lysate and mixed thoroughly. Both input and immunoprecipitation reaction were left to rotate overnight at 4°C.

The following day, immunoprecipitation samples were collected from the cold room and washed ten times with cold RIPA buffer (50 mM HEPES-KOH; 500 mM LiCl; 1 mM EDTA; 1% Igepal CA-630; 0.7% Na-deoxycholate) using a magnetic stand. On the last wash, samples were transferred to a fresh DNA LoBind tube and washed twice with cold TBS, before being resuspended in 200 µl of elution buffer (50 mM Tris-HCl; 10 mM EDTA; 1% SDS). Then 170 µl of elution buffer was added to the input samples and both were left at 65°C overnight on a shaker. The next day, supernatant was collected using a magnetic rack and all DNA samples were treated with RNaseA (Qiagen, 19101) and proteinase K before being purified using phenol chloroform extraction.

### ChIP-seq sequencing and analysis

Replicate input samples were pooled at an equimolar ratio and sample libraries were prepared using the NEBNext Ultra II library prep kit. Libraries were then pooled and sequenced paired end to 150 bp on a NovaSeq 6000. Adapter sequences were then trimmed using fastp (Chen et al., 2018) and reads mapped to the hg19 genome using Bowtie2 (Langmead and Salzberg, 2012). Narrow peaks were called using MACS2 (Zhang et al., 2008) with the DMSO input sample as the control and peaks with an enrichment score of less than four were removed. Differential peaks were identified using DESeq2 (Love et al., 2014) in DiffBind (https://bioconductor.org/packages/devel/bioc/vignettes/DiffBind/inst/doc/DiffBind.pdf) following reads in peaks library size normalisation using all peaks that were called in two out of three replicates for each sample. ChIPseeker (Yu et al., 2015) was used to annotate peaks to their nearest TSS within 50 kb unless otherwise stated. GO analysis was performed using clusterProfiler (Yu et al., 2012) using genes that showed an RLE normalised read count of >10 unless otherwise stated. Figures were made using ggplot2 (Wickham, 2009) in R (R Core Team, 2023).

### ChIP-qPCR

SK-N-BE(2)-C FUCCI reporter cells were collected and fixed with cold solution A as described above. After quenching in formaldehyde, cells were filtered and resuspended in FACS buffer (PBS with 5 mM EDTA; 25 mM HEPES: 0.1% BSA). Two million green cells (SG2M) and 4 million non-green cells (G0/G1) were sorted into FACS buffer and ChIP was performed as described above, including an IgG control. Four differentially bounds sites identified from ChIP-seq were tested using primers designed to amplify small (∼90 bp) regions. qPCRs were performed using PowerUp SYBR green master mix as per recommendations and run on an Applied Biosystems Stepone qPCR machine (see Table S1 for primer sequences).

### Generation of ASCL1 knockout line

This line was generated using the CRISPR-Cas9 system as previously described (Parkinson et al., 2022). The Cas9-2A-GFP and the U6-BsaI-sgRNA plasmids used for ASCL1 KO were kindly gifted by Prof. Steve Pollard (University of Edinburgh, UK), the design and generation of these plasmids has been previously described (Bressan et al., 2017) (see Table S1 for sgRNA sequences). Plasmids were transfected using Lipofectamine 2000 and cells were left to recover for 48 h. Clones were generated by FACS sorting single Cas9-GFP-positive cells followed by expansion. Two clones were used, harbouring a one base pair deletion (c.883delC) and a one base pair insertion (c.793_794insG), or a one base pair deletion (c.883delC) and a two base pair insertion (c.882_883insGC). Absence of ASCL1 was confirmed by western blot.

### Western blot

Cells were collected by scraping into 1 ml of ice-cold PBS and protein was extracted by resuspending the cell pellet in RIPA buffer plus cOmplete protease inhibitor and incubating for 20 min on ice. Debris was removed by centrifuging at 16,000 ***g*** for 10 min at 4°C and protein concentration was determined by BCA assay. An equal mass of protein was denatured in 1× NuPAGE LDS sample buffer with β-mercaptoethanol at 70°C for 10 min then loaded and separated on a BisTris gel with NuPAGE MOPS SDS running buffer at 150 V. Protein was transferred to a nitrocellulose membrane over 1 h at 4°C and 100 V then the membrane was blocked for 1 h using 5% milk. Primary antibodies were diluted in 1% milk (1:1000) and incubation took place overnight at 4°C. Membranes were washed thoroughly with Tris buffered saline with Tween-20 (TBST) and secondary antibodies (anti-mouse or anti-rabbit HRP conjugated whole antibodies) were diluted in TBST (1:5000) then incubated with membranes at room temperature for 1 h. Visualisation was performed using ECL western blotting detection reagent as per manufacturer's instructions before being exposed to X-ray film.

### Proliferation assay

Cell lines were plated in duplicate, and cells were quantified on a CellCountess II Automated Cell Counter, using Trypan Blue cell stain (Gibco, 15250061) to discriminate live and dead cell populations. Three biological replicates were measured per cell line.

### RNA-seq

ASCL1 wild-type SK-N-BE(2)-C and ASCL1 CRISPR knockout cells were plated at equal densities and treated with 1 µM of palbociclib (or vehicle) for 24 h or 7 days. Cells were lysed *in situ* using RLT buffer and RNA was purified using the RNeasy mini kit (Qiagen, 74104) as per manufacturer's instructions. Poly-A selection was performed, and libraries were made using NEBNext Ultra II directional RNA kit. Libraries for five biological replicates were pooled and 100 bp paired end reads were generated on a NovaSeq 6000 (Illumina). Reads were trimmed and quality filtered using TrimGalore with a minimum phred score of 20 then aligned to hg19 using STAR (Dobin et al., 2013) with quantMode to obtain read counts. DESeq2 (Love et al., 2014) was used to normalise read counts and identify differentially expressed genes.

### ATAC-seq

For ATAC-seq of cell cycle FACS sorted cells, asynchronous SK-N-BE(2)-C cells were plated in 15 cm^2^ dishes and grown to near confluency. Live cells were then stained with 3 μg ml^−1^ Hoechst 33342 for 1 h, before being trypsinised and collected. Cells were centrifuged for 5 min at 600 ***g*** before being resuspended in FACS buffer (PBS with 5 mM EDTA; 25 mM HEPES: 0.1% BSA) and filtered with a 45 µm filter. Then 50,000 G1 or G2M cells were sorted into 500 µl FACS buffer using a FACSariaIII Cell Sorter (BD Biosciences) based on UV 355 signal. For ATAC-seq of asynchronous parental SK-N-BE(2)-C cells and ASCL1 knockout cells, 50,000 live cells from the total population were sorted using a FACSariaIII. For all samples, ATAC-seq was performed using the Omni-ATAC protocol as previously described (Corces et al., 2017). Briefly, cells were pelleted and resuspended in 1 ml of cold resuspension buffer (RSB; 10 mM Tris-HCl pH 7.4, 10 mM NaCl, 3 mM MgCl_2_). Cells were spun at 500 ***g*** for 5 min at 4°C, then resuspended in 50 µl of RSB containing 0.1% Tween-20, 0.1% Igepal CA-630 and 0.01% digitonin and incubated on ice for 3 min. Then 1 ml of RSB containing only 0.1% Tween-20 was added and nuclei were centrifuged for 10 min at 500 ***g*** at 4°C. Nuclei were then resuspended in 50 µl of transposition mix [25 µl 2× TD buffer, 2.5 µl transposase (Illumina Tagment DNA Enzyme and Buffer kit, 20034198), 16.5 µl PBS, 0.5 µl 1% digitonin, 0.5 µl 10% Tween-20 and 5 µl H_2_O] and incubated at 37°C for 30 min. DNA was then cleaned and purified using the Zymo DNA Clean and Concentrator-5 kit before being amplified and quantified on a Agilent tapestation. Three biological replicates of cell cycle sorted cells were prepared, pooled and sequenced on a NovaSeq 6000 to generate 100 bp paired end reads. Four replicates of the asynchronous parental and ASCL1 knockout cells were prepared, pooled and sequenced on a NovaSeq 6000 to generate 50 bp paired end reads. Resulting reads were trimmed using TrimGalore with a Phred score cut-off of 20 and then mapped to hg19 using Bowtie2 (Langmead and Salzberg, 2012). MACS2 (Zhang et al., 2008) was used to call peaks and DiffBind (https://bioconductor.org/packages/devel/bioc/vignettes/DiffBind/inst/doc/DiffBind.pdf) (EdgeR; Robinson et al., 2010) for identifying differential peaks with reads in peaks library size normalisation. Bigwigs were scaled according to these scaling factors.

### Imaging

Fluorescent imaging was performed on an Olympus IX51 inverted microscope, while phase contrast only images were taken using an EVOS M5000.

### Immunocytochemistry and flow cytometry

SK-N-BE(2)-C FUCCI reporter cells were cultured to near confluency before being trypsinised and collected. Cells were fixed for 10 min in 1 ml of 4% formaldehyde followed by quenching with 110 µl of 1.25 M glycine. Cells were then spun down and washed in FACS buffer (PBS with 5 mM EDTA, 25 mM HEPES, 0.1% BSA) before being resuspended in FACS buffer plus Triton X-100 0.2% (FBT) for 10 min with rotation. Non-specific sites were blocked with PBS plus 3% BSA for 30 min with rotation. Cells were spun down then resuspended in 1 ml FBT plus 2% FBS with 1.25 µg of antibody (ASCL1, ab211327; TBP, 22006-1-AP). Cells were washed once in FBT then resuspended in 500 µl of FBT, 2% FBS plus 2 µl of the relevant secondary antibody (donkey anti-rabbit 647 or mouse anti-rabbit 647) and 0.5 µl of Hoechst 33342. Cells were rotated in the dark for 30 min then washed once with FBT and once with FACS buffer and filtered with a 45 µm filter. Samples were run on a BD LSRFortessa flow cytometer and cell cycle phases gated based on UV 355 nm signal intensity.

### Quantification and statistical analysis

Statistical analyses were performed using Prism or R software (R Core Team, 2023), with each statistical test, sample sizes and significance thresholds noted in the figure legends. Biological replicates were derived from cells at different passage numbers. Statistical analysis of differential binding and differential accessibility was performed using DiffBind (https://bioconductor.org/packages/devel/bioc/vignettes/DiffBind/inst/doc/DiffBind.pdf) in R and statistical analyses of RNA-seq data were performed using DESeq2 (Love et al., 2014) in R (R Core Team, 2023). We used Bonferroni's multiple testing correction in Fig. 5B,C, whereby the new significance level is obtained by dividing the canonical *P*-value of 0.05 by the number of statistical tests (seven in each case).

## Supplementary Material



10.1242/develop.204816_sup1 Supplementary information
