# Supplementary material for: Distinct proliferative and neuronal programmes of chromatin binding and gene activation by ASCL1 are cell cycle stage-specific
Source: Development. 2025 Jun 25;152(12):dev204816. doi: 10.1242/dev.204816 (PMC12273627; doi:10.1242/dev.204816)
Supplement: Supplementary information [file develop-152-204816-s1.pdf]

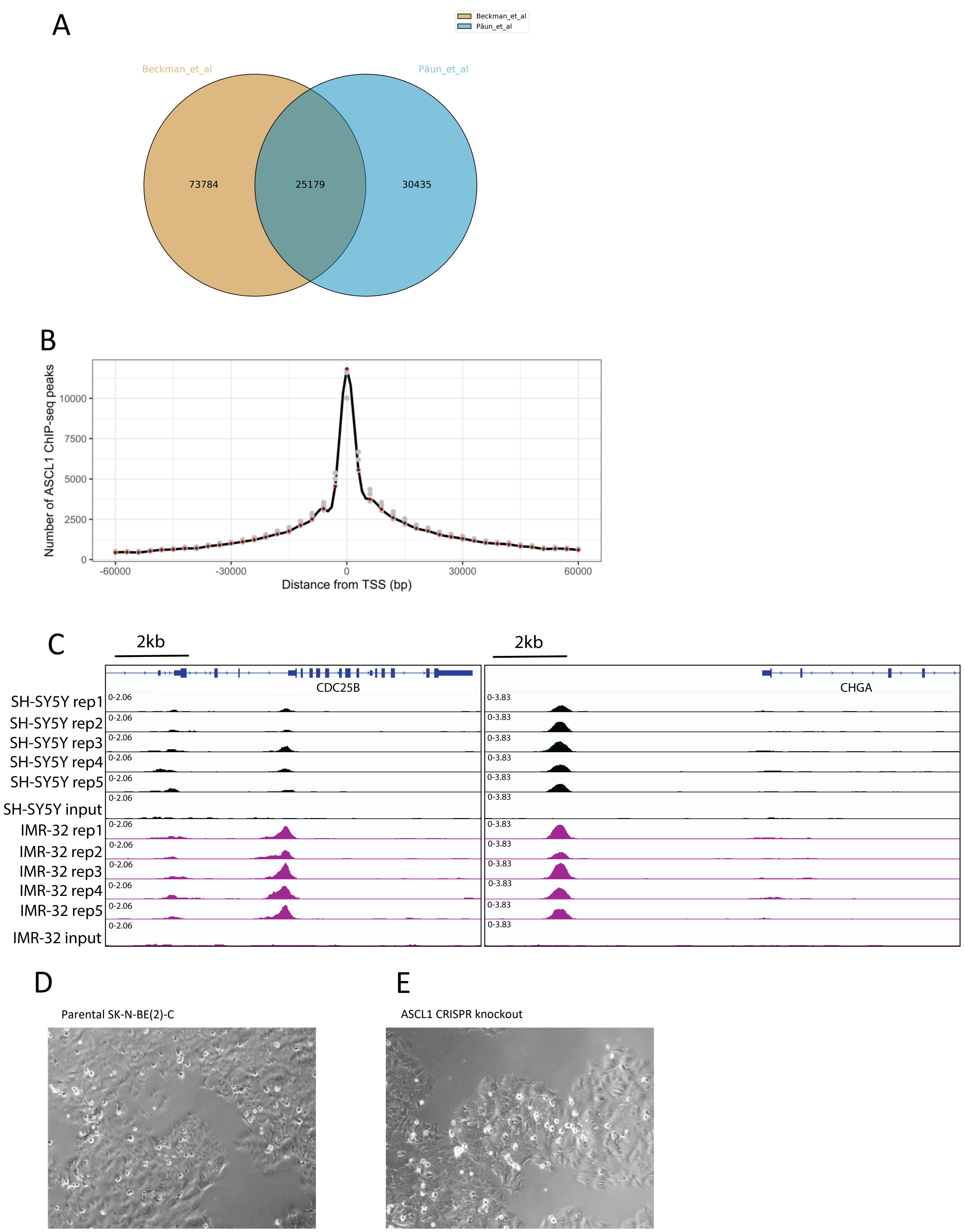

Development • Supplementary information

Fig. S1. ASCL1 binds to both proliferative genes and neuronal genes in asynchronous cell populations. (A) Intersection between consensus ASCL1 ChIP-seq peaks in asynchronous SK-N-BE(2)-C cells (orange, this study) and cortical neurons previously described (blue) (Păun *et al.*, 2023). (B) Peak locations from 3 SK-N-BE(2)-C ASCL1 ChIP-seq replicates (grey) binned into 3kb windows from gene TSSs and quantified. Black line depicts the spline for the average across the replicates (red). (C) ASCL1 ChIP-seq tracks of five biological replicates in asynchronous SH-SY5Y cells (black) and IMR-32 cells (purple), plus the input controls. Regions around the CDC25B and CHGA TSSs are shown. (D) Phase contrast image of parental SK-N- BE(2)-C cell line. (E) Phase contrast image of the ASCL1 CRISPR knock-out clone.

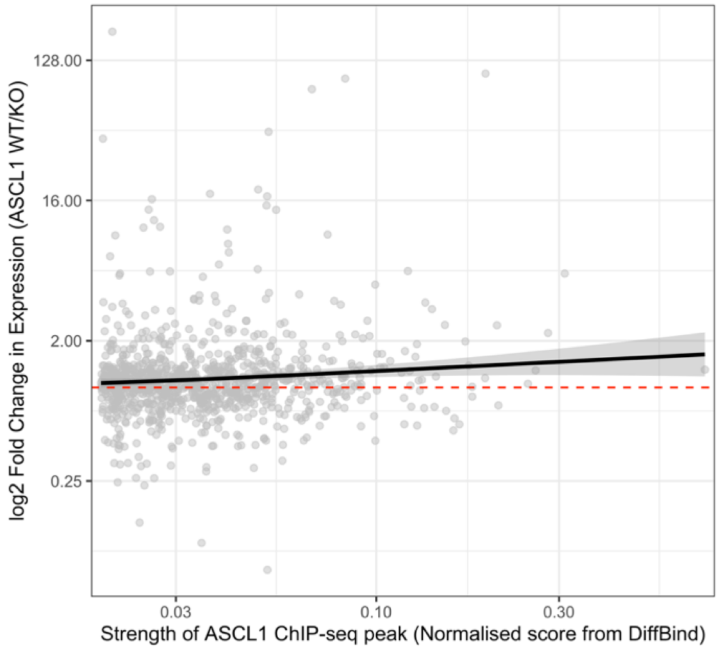

Fig. S2. ASCL1 knock-out leads to a general reduction in expression level of bound genes 1000 genes associated with the strongest ASCL1 peaks (normalised peak score from DiffBind) were plotted against their fold change in expression following ASCL1 knock-out. The local polynomial regression (LOESS, black line) is consistently greater than 1 (red dotted line; no fold change).

Development • Supplementary information

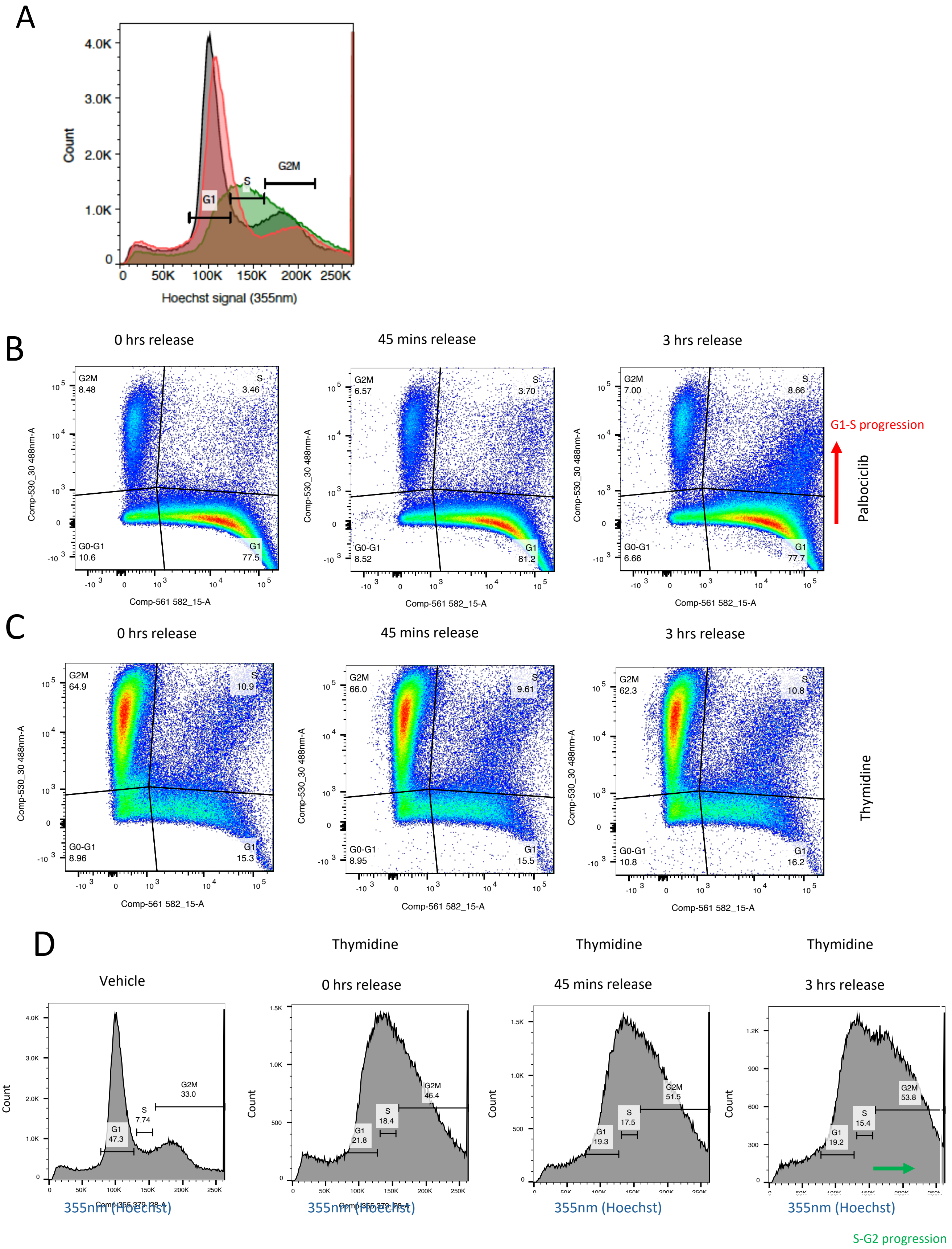

Development • Supplementary information

Fig. S3. Cell cycle synchronisation is effective and reversible. (A) Flow cytometry plot of SK-N-BE(2)-C FUCCI cell line treated with DMSO (black), palbociclib (red) or thymidine (green), stained with Hoechst 33342. Cells are gated based on 355 nm signal into G1 phase, S phase and G2M phase. (B) Flow cytometry and cell cycle analysis based on FUCCI fluorophores for cells treated with palbociclib followed by wash out for 0 min (left), 45 min (middle) and 3 hr (right). X-axis shows mKO2 signal while y-axis shows mAG signal. (C) Flow cytometry and cell cycle analysis based on FUCCI fluorophores for cells treated with thymidine followed by wash out for 0 min (left), 45 min (middle) and 3 hr (right). X-axis shows mKO2 signal while y-axis shows mAG signal. (D) Flow cytometry and cell cycle analysis based on DNA content for DMSO 23 treated cells (left) and cells treated with thymidine followed by wash out for 0 min (centre left), 45 min (centre right) and 3 hr (right). The same gates were used for all plots.

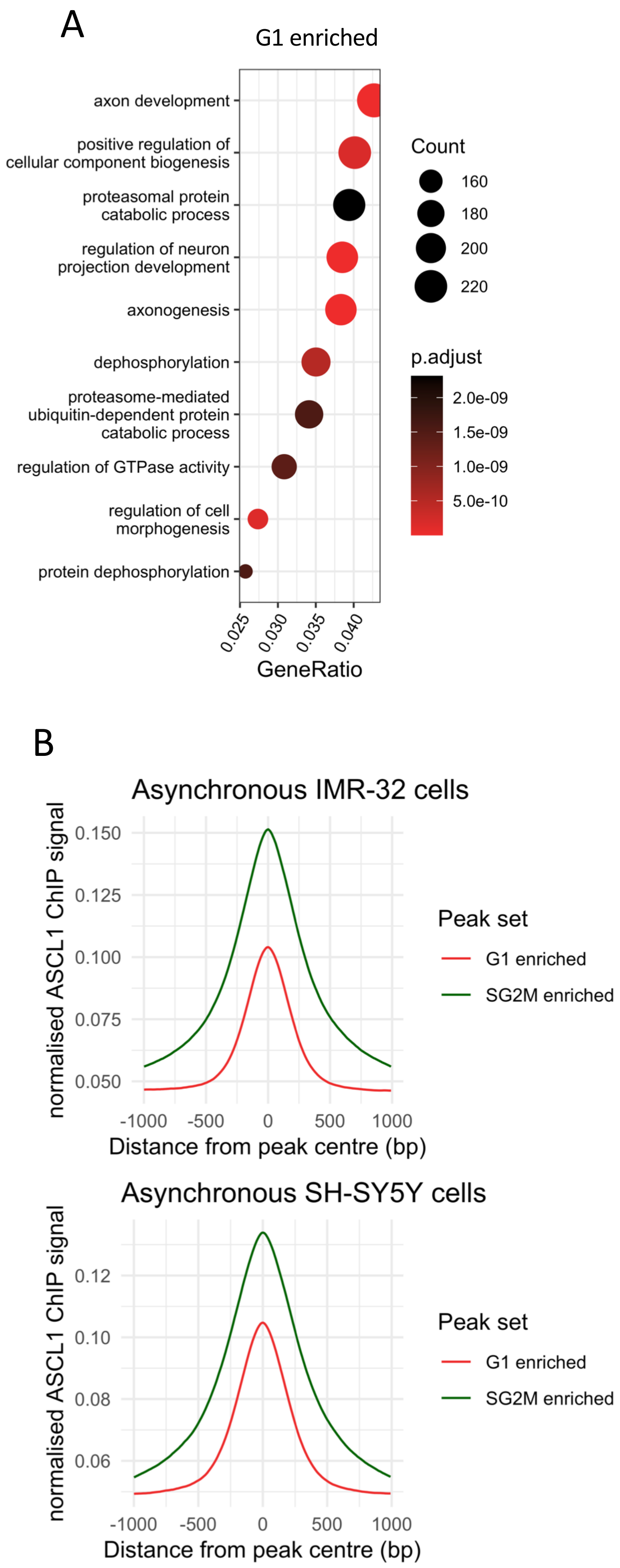

Development • Supplementary information

Fig. S4. ASCL1 binding dynamics are consistent in additional neuroblastoma cell lines. (A) Gene ontology analysis for ASCL1 bound, G1 enriched sites (red, left) and SG2M enriched sites (green, right) when peaks are linked to the nearest TSS, irrespective of distance). (B) Normalised ASCL1 ChIP-seq signal for peaks showing enriched ASCL1 binding in G1 phase (red) and SG2M phase (green) in asynchronous IMR-32 cells (top) and SH-SH5Y cells (bottom).

Development • Supplementary information

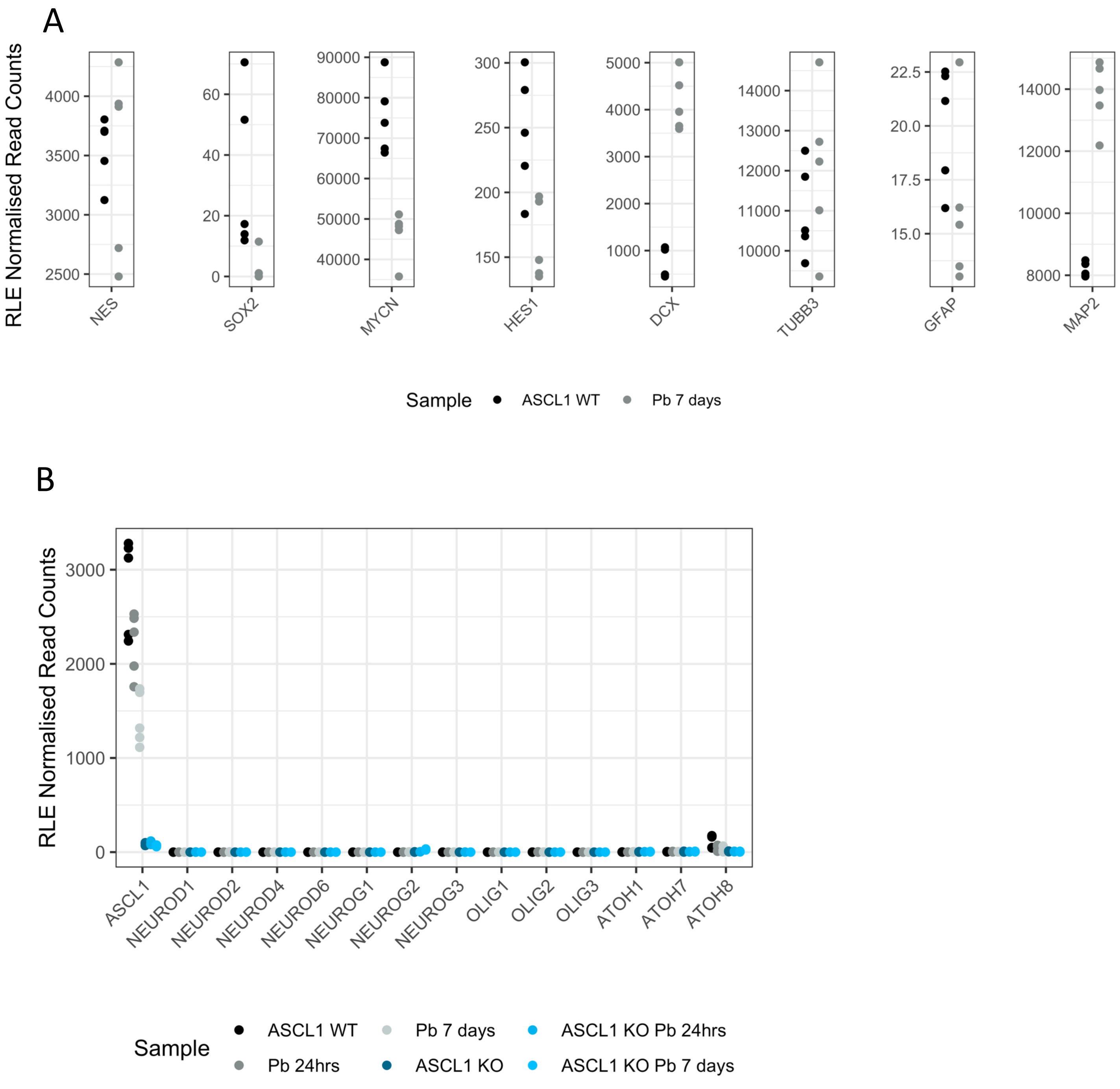

Fig. S5. Expression of specific key genes following palbociclib treatment of SK-N-BE(2)-C cells (A) Normalised RNA expression of stemness markers (NES, SOX2, MYCN, HES1) and neuronal markers (DCX, TUBB3, GFAP, MAP2) in ASCL1 WT cells before (black) and after (grey) 7 days palbociclib treatment. (B) Normalised RNA expression of ASCL1 and other key proneural transcription factors in ASCL1 WT cells (black, greys) and ASCL1 KO cells (blues) before and after palbociclib treatment.

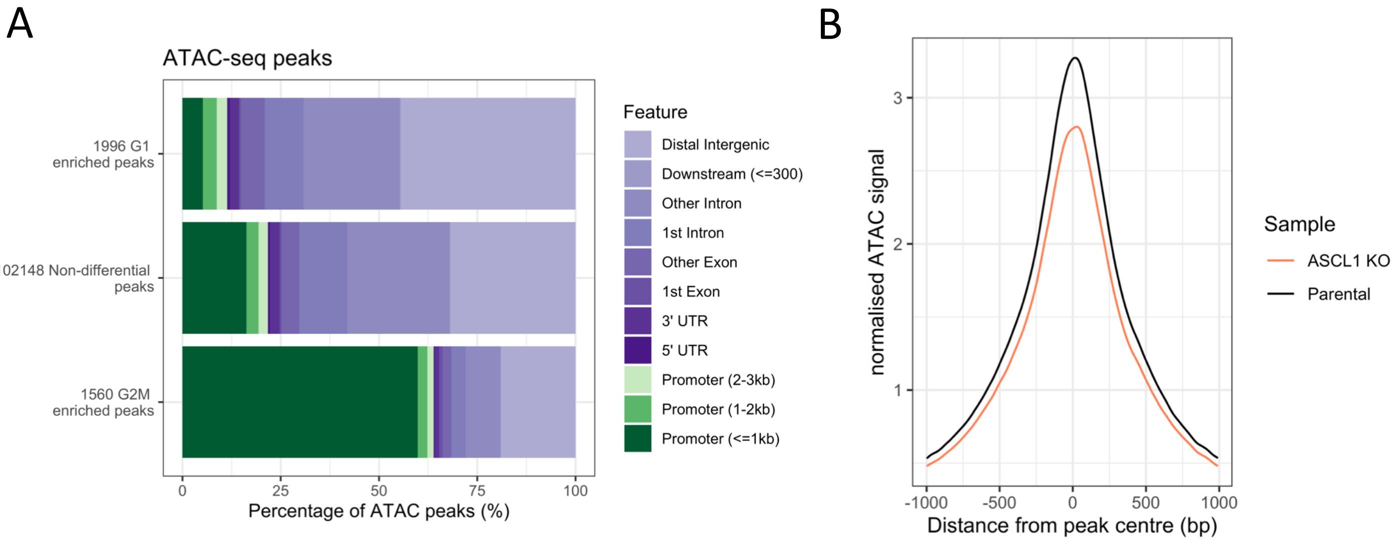

Fig. S6. G1 enriched accessibility is associated with distal regulatory elements, while G2M enriched accessibility is associated with promoter elements. (A) G1 enriched (top), G2M enriched (bottom) and non-differential (middle) ATAC-seq peaks from FACS sorted populations. Peaks are coloured based on their genomic locations relative to known genes (green for promoter regions, purple for non-promoter regions). (B) Normalised ATAC-seq signal for parental SK-N-BE(2)-C (orange) and ASCL1 knock-out (black) asynchronous cells at ASCL1 bound, SG2M enriched peaks harbouring a high confidence canonical ASCL1 E- box motif.

Table S1. Oligonucleotide sequences used in the study.

Name	Sequence (5’ -> 3’)
ASCL1 CRISPR sgRNA_1	CGCTGTCGCTTGUCTTGCTT
ASCL1 CRISPR sgRNA_2	UUCTGUTGCGCTGCUUUCGC
EFNA5 ChIP-qPCR Forward	GAGCAAGACCAGCGAAATGG
EFNA5 ChIP-qPCR Reverse	AGTGAGTGAAGCTAGCAGCC
PDZD2 ChIP-qPCR Forward	GGGGAAGCCAGCTTCTAGTG
PDZD2 ChIP-qPCR Reverse	CTGCAGCTGGTCTCATCACA
CCNA2 ChIP-qPCR Forward	GCGGCTGTTCTTGCAGTTC
CCNA2 ChIP-qPCR Reverse	TGGTTTACCCTTCACTCGCC
GAS6 ChIP-qPCR Forward	GGGAACCACCAAAGTGGACA
Gas6 ChIP-qPCR Reverse	CCCCAGGATCTGGAGAGTGA
Geminin-pLenti (In-Fusion mAG-geminin cloning)	GAGGTTGATTGTCGATTACAGCGCCTTTCTCCG
pLenti-mAG (In-Fusion mAG-geminin cloning)	CGACTCTAGAGGATCTAATTCGCCACCATGGTGAGCG
CDT1-pLenti (In-Fusion mKO2-cdt1 cloning)	GAGGTTGATTGTCGAAGATTAGATGGTGTCCTGGTCC
pLenti-mKO2 (In-Fusion mKO2-cdt1 cloning)	CGACTCTAGAGGATCATTCGCCACCATGGTGAGTGT

Development • Supplementary information

Table S2. Key resources table

REAGENT or RESOURCE	SOURCE	IDENTIFIER
**Antibodies**		
ASCL1 (ChIP-seq)	Abcam	ab74065
ASCL1 (WB)	Abcam	ab211327
IgG (ChIP-qPCR)	Abcam	ab171870
α-Tubulin (WB)	ProteinTech	66031
Secondary antibody: Sheep anti-mouse IgG HRP conjugated whole Ab	GE Healthcare	NA931
Secondary antibody: Donkey anti-rabbit IgG HRP conjugated whole Ab	GE Healthcare	NA934
**Bacterial and virus strains**		
Stellar competent cells	Takara Bio	636763
**Biological samples**		
**Chemicals, peptides, and recombinant proteins**		
DMEM/F12	Sigma-Aldrich	D8437
IMDM	Gibco	21980032
FBS	Pan BioTech	P40-37500
Pen/Strep	Sigma-Aldrich	P0781
Trypsin EDTA 0.25%	Gibco	25200072
DMSO	Santa Cruz Biotechnology	sc-358801
BamH1-HF	New England Biolabs	R3136S
Sal1-HF	New England Biolabs	R3138S
Agarose	Sigma-Aldrich	A9539
Gel red 1000x stain in DMSO	Biotium	41002
6x purple loading dye	New England Biolabs	B7024S
DNA ladder	New England Biolabs	N0550S
Polybrene	Sigma-Aldrich	TR-1003-G
Palbociclib	Sigma-Aldrich	PZ0383
Thymidine	Sigma-Aldrich	T9250
Formaldehyde	VWR chemicals	20909.290
HEPES	Sigma-Aldrich	H3375

NaCl	ThermoFisher Scientific	BP358-1
EDTA	Millipore	324503
EGTA	Millipore	324626
Glycine	ThermoFisher Scientific	G/0800/60
cOmplete protease inhibitor	Roche	11836170001
Dynabeads Protein G	Invitrogen	10003D
BSA	Fisher BioReagents	BP9706-100
KOH	Sigma-Aldrich	757551
Glycerol	ThermoFisher Scientific	G/0600/08
Igepal CA-630	MP Bio	198596
Triton X-100	Sigma-Aldrich	X100
Trizma base	Sigma-Aldrich	T1503
HCl	Honeywell	72033
Na-Deoxycholate	Sigma-Aldrich	D6750
N-lauroylsarcosine	Sigma-Aldrich	L5777
Histone H2B	New England Biolabs	M2505S
Total RNA	ThermoFisher Scientific	4307281
LiCl	ThermoFisher Scientific	L/2201/50
Proteinase K	Invitrogen	EO0491
Phenol:chloroform:isoamylalcohol	Sigma-Aldrich	P3803
Ethanol	Sigma-Aldrich	32221-2.5L-M
RIPA buffer	Sigma-Aldrich	R0278
BisTris gel	Invitrogen	NP0301
Skimmed milk powder	Serva	42590
Tween-20	Promega Corporation	H5151
Nitrocellulose membrane	Bio-Rad	1620115
NuPAGE MOPS SDS running buffer	Invitrogen	NP0001
NuPAGE LDS buffer	Invitrogen	NP0007
β-mercaptoethanol	BDH	441433A
Hoechst 33342	Invitrogen	H3570
$MgCl_2$	Sigma-Aldrich	M1028
Digitonin	Sigma-Aldrich	D141
PowerUp™ SYBR™ Green Master Mix	Applied Biosystems	A25742
Methanol	ThermoFisher Scientific	M/4056/15

Development • Supplementary information

**Critical commercial assays**		
ECL Western Blotting Detection Reagents	Cytivia	RPN2106
CellCountess II Automated Cell Counters	ThermoFisher Scientific	C10312
RNeasy mini kit	QIAGEN	74104
NEBNext Ultra II directional RNA kit	New England Biolabs	E7760L
Lipofectamine 2000	ThermoFisher Scientific	11668019
Tagment DNA enzyme and buffer kit	Illumina	20034198
DNA Clean and Concentrator-5 kit	ZYMO	D4014
D1000 Tapestation reagents	Agilent	5067
QuantiTect reverse transcription kit	Qiagen	205311
BCA protein assay kit	ThermoFisher Scientific	23227
Plasmid Maxi kit	QIAGEN	12162
**Deposited data**		
ASCL1 ChIP-seq; raw and analysed data	This manuscript	GSE276842
Cell cycle sorted ATAC-seq; raw and analysed data	This manuscript	GSE276994
ASCL1 KO ATAC-seq; raw and analysed data	This manuscript	GSE276994
RNA-seq; analysed data	This manuscript	GSE276843
Original code	This manuscript	DOI:10.5281/zenodo.15601707
Neuroblastoma survival data	R2 Genomics Platform	Tumour Neuroblastoma public - Versteeg - 88 MAS5.0 - u133p2
**Experimental models: Cell lines**		
SK-N-BE(2)-C cells	Laboratory of Prof. Deborah Tweedle, Newcastle University	N/A
HEK293T cells	Cambridge Stem Cell Institute Tissue Culture facility	N/A
IMR-32 cells	Laboratory of Prof. Deborah Tweedle, Newcastle University	N/A

Development • Supplementary information

SH-SY5Y cells	Laboratory of Prof. John Hardy, UCL	N/A
**Experimental models: Organisms/strains**		
**Oligonucleotides**		
ChIP-qPCR primer sequences	Sigma-Aldrich	N/A
ASCL1 CRISPR sgRNA sequences	Laboratory of Prof. Steve Pollard, University of Edinburgh	N/A
In-Fusion primer sequences for FUCCI reporter cloning	Sigma-Aldrich	N/A
**Recombinant DNA**		
pCAG-mKO2-cdt1	Laboratory of Prof. Ludovic Vallier	N/A
pCAG-mAG-gem	Laboratory of Prof. Ludovic Vallier	N/A
pLenti-CMV-mAG-gem	This study	N/A
pLenti-CMV-mKO2-cdt1	This study	N/A
**Software and algorithms**		
FIJI/Image J	Open Source	https://imagej.net/software/fiji/
GraphPad Prism version 10.3.0	GraphPad	https://www.graphpad.com/scientific-software/prism/
StepOne Software	ThermoFisher Scientific	https://www.thermofisher.com/uk/en/home/technical-resources/software-downloads/StepOne-and-StepOnePlus-Real-Time-PCR-System.html

TapeStation Software	Agilent	https://www.agilent.com/en/product/automated-electrophoresis/tapestation-systems/tapestation-software
IGV v2.17.4	Robinson et al. (Robinson *et al.*, 2011)	https://igv.org/
R Studio v 2024.04.0+735	CRAN (R Core Team, 2023)	https://cran.r-project.org/
ChIPpeakAnno v3.38.0	Zhu et al. (Zhu *et al.*, 2010)	https://bioconductor.org/packages/release/bioc/html/ChIPpeakAnno.html
edgeR v4.2.0	Robinson et al. (Robinson, McCarthy and Smyth, 2010)	https://bioconductor.org/packages/release/bioc/html/edgeR.html
Rtracklayer v1.64.0	Lawrence et al. (Lawrence, Gentleman and Carey, 2009)	https://www.bioconductor.org/packages/release/bioc/html/rtracklayer.html
DiffBind v3.14.0	Stark et al. (Stark and Brown, 2011)	https://bioconductor.org/packages/release/bioc/html/DiffBind.html
ChIPseeker v1.40.0	Yu et al. (Yu, Wang and He, 2015)	https://github.com/YuLab-SMU/ChIPseeker
TxDb.Hsapiens.UCSC.hg19.knownGene v3.2.2	Bioconductor	https://bioconductor.org/packages/release/data/annotation/html/TxDb.Hsapiens.UCSC.hg19.knownGene.html

Development • Supplementary information

DESeq2 v1.44.0	Love et al. (Love, Huber and Anders, 2014)	https://bioconductor.org/packages/release/bioc/html/DESeq2.html
clusterProfiler v4.12.0	Yu et al. (Yu *et al.*, 2012)	https://github.com/YuLab-SMU/clusterProfiler
org.Hs.eg.db v3.19.1	Bioconductor	https://bioconductor.org/packages/release/data/annotation/html/org.Hs.eg.db.html
Enrichplot v1.24.0	Bioconductor	https://www.bioconductor.org/packages/release/bioc/html/enrichplot.html
ggpubr v0.6.0	CRAN	https://cran.r-project.org/web/packages/ggpubr/index.html
ggplot2 v3.5.1	Wickham et al. (Wickham, 2009)	https://cran.r-project.org/web/packages/ggplot2/index.html
Tidyverse v2.0.0	CRAN	https://cran.r-project.org/web/packages/tidyverse/index.html
RColorBrewer v1.1-3	CRAN	https://cran.r-project.org/web/packages/RColorBrewer/index.html
EnrichedHeatmap v1.34.0	Gu et al. (Gu *et al.*, 2018)	https://bioconductor.org/packages/release/bioc/html/EnrichedHeatmap.html

Development • Supplementary information

TrimGalore v0.6.10	Babraham Bioinformatics	https://github.com/FelixKrueger/TrimGalore
STAR v2.6.1a	Dobin et al. (Dobin *et al.*, 2013)	https://github.com/alexdobin/STAR
Bowtie2 v2.5.4	Langmead et al.	https://bowtie-bio.sourceforge.net/bowtie2/manual.shtml
MACS2 v2.2.7.1	Zhang et al.	https://github.com/macs3-project/MACS
fastp v0.23.4	Chen et al.	https://github.com/OpenGene/fastp
**Other**		
X-ray film	Scientific Laboratory Supplies	MOL7016

Development • Supplementary information
