# Supplementary material for: Distinct proliferative and neuronal programmes of chromatin binding and gene activation by ASCL1 are cell cycle stage-specific
Source: Development. 2025 Jun 25;152(12):dev204816. doi: 10.1242/dev.204816 (PMC12273627; doi:10.1242/dev.204816)
Supplement: Supplementary file 1 [file develop-152-204816_review_history.pdf]

Distinct proliferative and neuronal programmes of chromatin binding and gene activation by ASCL1 are cell cycle stage-specific

William F. Beckman, Lydia Parkinson, Lewis Chaytor and Anna Philpott
DOI: 10.1242/dev.204816

Editor: Francois Guillemot

Review timeline
Submission to Review Commons: 17 November 2024
Submission to Development: 27 March 2025
Editorial decision: 22 April 2025
First revision received: 20 May 2025
Accepted: 23 May 2025

Reviewer 1

Evidence, reproducibility and clarity

This is an interesting study investigating the role of the proneural transcription factor ASCL1 in neuroblastoma. Previous work has shown that over-expression of ASCL1 can drive differentiation on neuroblastoma cells, but the gene also has roles in maintaining proliferation. The authors carry out a series of genomic studies including ChIP-seq and ATAC-seq to untangle these different roles of ASCL1. While most of the work presented is well-done and the analysis is straightforward, there are some concerns with the conclusions, since some key controls have not been done.

1. The authors have not done a motif analysis of the ASCL1-ChIPseq so it is not clear whether E-box motifs are enriched/dominate. This is an important control. Also, it would be very useful to compare the ASCL1-ChIP-seq with other published datasets in other neural tissues, as an additional control.
2. Most of the analysis is done on regions that are less than 50 kb from the nearest TSS. This restricts the analysis to about half the peaks. Since they observe a difference between the G2M peak and the G1 peaks in their distance from the TSS< it would be useful to show whether the same relationship holds when all peaks are included. This may stregthen the finding.
3. The correlate the genes that decline with ASCL1 KO and the peaks from the ChIP-seq using GO terms, but would be very useful to determine how many of these genes are direct targets. This can bve done by showing the correlaiton between the RNAseq and the ChIP- seq on a gene-by-gene basis rather than using GO.
4. The cell cycle synchronization experiments are a good confirmation of the unsynchronized experiments.
5. The lack of ASCL1 dependence of the G1 neuronal genes (Fig 5B) is interesting, but may be confounded by the possibility that these sites are driven equally well by a redundant proneural trnascription factor, like NEUROD1 or NEUROG. This possibility should be addressed by carrying out ChIP for these factors at select sites (G2M vs G1). Alternatively ChIP-seq for these factors would be ideal. Without these experiments the conclusion is not supported: "This indicates that ASCL1 is capable of binding to neuronal targets in G1 phase of the cell cycle in neuroblastoma cells but is not supporting their expression under cycling conditions."
6. The problem of redundant TFs is also an issue with the experiments to teat the effects of long G1 arrest.
7. The finding that G1 ASCL1 sites show less accessibility than G2M sites is interesting; is thre a reduction in ASCL1 ChIP-seq signal at these sites as well? Or is ASCL1 bound but not able to open the chromaitn at these sites?

© 2023. Published by The Company of Biologists under the terms of the Creative Commons Attribution License (https://creativecommons.org/licenses/by/4.0/).

8. The reduction in accessible sites in the ASCL1 KO for the G2M sites is consistent with the effects on proliferation, but the effect is very modest. Would this effect be greater if the analysis of the ATAC-seq data were confined to sites with E-boxes? it would be useful to know what percentage of the accessible sites have an E-box and what percent of these sites are lost in the ASCL1 KO. This might show the importance of redundant proneural TFs.

Significance

This is an interesting study and provides new insight into the dual mechanisms of proneural transcription factors in neuroblastoma proliferation and differentiation. Since ASCL1 has similar dual roles in proliferation and neural differentiation in normal CNS development, the results of this report will improve the understanding of this factor more generally.

Reviewer 2

Evidence, reproducibility and clarity

In this manuscript by Beckman et al. the authors propose that different dynamics of Ascl1 binding to promoters of cell cycle and neuronal genes could explain the known association between cell cycle lengthening and differentiation. This stems from their observation that Ascl1 binds preferentially enhancers of neuronal genes in G1, although it does not drive their expression, while it binds the promoters and regulates the expression of cell cycle associated genes in G2/M. They also show that lengthening of G1 through pharmacological means increases chromatin accessibility (shown by ATAC-seq and H3K27ac) and allows Ascl1 to induce the expression of neuronal genes. They therefore propose a system where Ascl1 binds to primed neuronal enhancers in G1 but only drives their expression when a lengthened G1 phase has previously allowed chromatin changes involving histone modification. Their data is nicely controlled using Asc1cKO cells, allowing them to show specificity to the ability of Ascl1 to promote the expression of neuronal vs cell cycle genes. Overall, the work is nicely executed and clearly presented.

There is however, one important concern to be clarified before strong conclusions can be extracted from the data: are palbociclib-treated cells comparable to control cells? 7 days of G1 arrest could have led to differentiation of at least a fraction of the NSCs and therefore the increased expression of neuronal genes (and chromatin changes) could reflect a higher percentage of differentiated cells (or higher degree of differentiation) in that sample rather than increased expression of neuronal genes in NSCs. A characterization of the cultures after the 7-day treatment is therefore necessary before drawing any conclusions. This could be done through immunohistochemistry to assess the presence of differentiated cells and control for the continuous and homogeneous expression of stemness markers (some useful markers include Nestin, Sox2, DCX, Tubb3 or GFAP). The reversibility assay, as shown in Figure S2 would also be very informative for the 7-day time point.

Other minor points:

- In figure 2, it would be interesting to display the overlap between bound and regulated genes.
- Please clarify where does the number of 47,294 non-commonly regulated genes between G1 and S/G2/M come from. From the data in figure 3D the number should be roughly 30k.
- In figure 3F/G, it would be very informative to show also examples of cell cycle independent genes.
- In graph 4B, please unify the way the legend is displayed (location of "count" and "p.adjust").
- In figure 5A, could it be that the expression levels of neuronal genes are too low in control cells, so that it is difficult to see a difference in the cKO cells? Even if not significant, would be good to show the p value.
- And simply a style point: I found the color scheme for significance in the graphs confusing, as dark colors signify less significance and white/clear shades high significance.

Significance

© 2023. Published by The Company of Biologists under the terms of the Creative Commons Attribution License (https://creativecommons.org/licenses/by/4.0/).

This work addresses an important long-standing question: how can Ascl1 simultaneously promote cell cycle and neurogenesis? It will be of relevance for the fields of neurogenesis, stem cell biology, reprogramming, and cancer biology.

Conceptually, it could be made clearer in the discussion that Ascl1 appears to be dispensable for the increased chromatin accessibility caused by G1 lengthening, and even for the expression of neuronal genes (as shown in figure 5B, where there is a similar increase in neuronal genes expression in the absence of Ascl1 than in control cells after 7 days of palbociclib). This won't compromise the significance of the work, which has the potential to explain the dual role of Ascl1 in NSCs. But will hopefully encourage the field to further investigate the mechanisms behind the effects of G1 lengthening on chromatin accessibility.

Author response to reviewers' comments

1. General Statements [optional]

This section is optional. Insert here any general statements you wish to make about the goal of the study or about the reviews.
Our manuscript shows that, in cycling cells, the proneural master regulator transcription factor ASCL1 binds preferentially to pro-neurogenic enhancers in G1 phase of the cell cycle but this binding does not drive gene expression. As cells move to S/G2, ASCL1 binding is now enriched at promoters of pro- proliferative genes where it activates gene expression to maintain a pro-proliferative progenitor state. However, stalling of the cell cycle in G1 allows ASCL1 binding at enhancers to facilitate H3K27ac deposition and pro-neurogenic gene expression, driving the differentiation programme. We thus show hitherto unknown cell cycle dependency of distinct transcriptional programmes driven by the same transcription factor at different cell cycle stages and reveal why a lengthening specifically of G1 can allow engagement of a differentiation programme by turning unproductive factor binding into a productive interaction.

We note, Reviewer 1:
This is an interesting study and provides new insight into the dual mechanisms of proneural transcription factors in neuroblastoma proliferation and differentiation. Since ASCL1 has similar dual roles in proliferation and neural differentiation in normal CNS development, the results of this report will improve the understanding of this factor more generally.
from Reviewer 2:
This work addresses an important long-standing question: how can Ascl1 simultaneously promote cell cycle and neurogenesis? It will be of relevance for the fields of neurogenesis, stem cell biology, reprogramming, and cancer biology.

We thank the reviewers for their very positive evaluations of the paper and its implications. Where questions and concerns were raised we have addressed them fully, below.

2. Point-by-point description of the revisions

This section is mandatory. Please insert a point-by-point reply describing the revisions that were already carried out and included in the transferred manuscript.

Reviewer 1:
"The authors have not done a motif analysis of the ASCL1-ChIPseq so it is not clear whether E-box motifs are enriched/dominate. This is an important control. Also, it would be very useful to compare the ASCL1-ChIP-seq with other published datasets in other neural tissues, as an additional control."

Prompted by this comment, we have performed motif analysis on the consensus set of ASCL1 ChIP-seq peaks in the DMSO control samples (i.e. freely cycling cells). This identified the

© 2023. Published by The Company of Biologists under the terms of the Creative Commons Attribution License (https://creativecommons.org/licenses/by/4.0/).

canonical ASCL1 E-box motif as the most significantly enriched, occurring in the majority of peaks: We have now added this motif analysis output to Figure 1A.

Total Target Sequences = 98970, Total Background Sequences = 97333

Rank	Motif	Name	P-value	log P-pvalue	q-value (Benjamini)	# Target Sequences with Motif	% of Targets Sequences with Motif
1	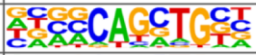	Ascl1(bHLH)/NeuralTubes-Ascl1-ChIP-Seq(GSE55840)/Homer	1e-8767	-2.019e+04	0.0000	50564.0	51.09%
2	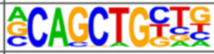	Tcf12(bHLH)/GM12878-Tcf12-ChIP-Seq(GSE32465)/Homer	1e-8188	-1.885e+04	0.0000	37684.0	38.08%
3	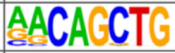	MyoG(bHLH)/C2C12-MyoG-ChIP-Seq(GSE36024)/Homer	1e-8082	-1.861e+04	0.0000	37809.0	38.21%
4	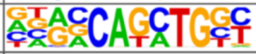	Atoh1(bHLH)/Cerebellum-Atoh1-ChIP-Seq(GSE22111)/Homer	1e-7892	-1.817e+04	0.0000	37678.0	38.07%
5	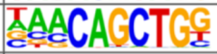	Tcf21(bHLH)/ArterySmoothMuscle-Tcf21-ChIP-Seq(GSE61369)/Homer	1e-7838	-1.805e+04	0.0000	35516.0	35.89%
6	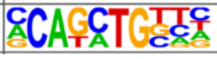	Twist2(bHLH)/Myoblast-Twist2.Ty1-ChIP-Seq(GSE127998)/Homer	1e-7779	-1.791e+04	0.0000	48091.0	48.59%

As requested, we downloaded a previously published ASCL1 ChIP-seq dataset (Păun et al. 2023) where human iPSCs were differentiated into cortical neurons. We find that ~25% of our consensus peakset intersects with binding sites detected in cortical neurons, representing just under 50% of this latter set. This is a large intersection of 25,000 peaks, especially considering the developmental differences between the two cell types (neuroblastic progenitors of the PNS versus more differentiated cortical neurons of the CNS). We have now added this figure to Supplementary Figure 1.

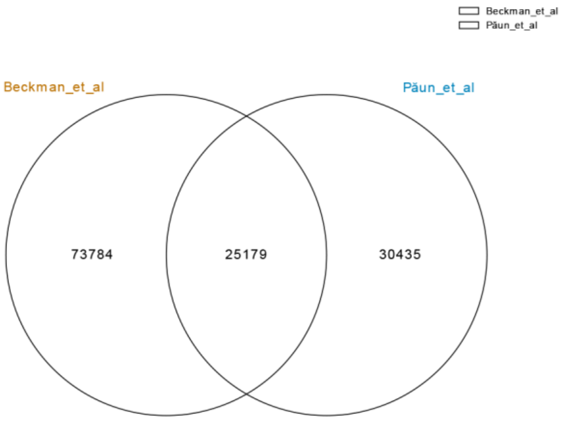

“Most of the analysis is done on regions that are less than 50 kb from the nearest TSS. This restricts the analysis to about half the peaks. Since they observe a difference between the G2M peak and the G1 peaks in their distance from the TSS< it would be useful to show whether the same relationship holds when all peaks are included. This may stregthen the finding.”

Our ChIP-seq protocol was very sensitive and detected even low levels/transient ASCL1 binding, giving a large number of ASCL1 peaks. Consequently, a significant fraction of the genes in the genome became associated with ASCL1 binding and so we used a stringent distance based cut-off based on the assumption that there is a higher likelihood of enhancers acting on nearby promoters, rather than those further away. When we link all peaks to their nearest TSS, irrespective of distance, we find a similar trend, namely G1 enriched ASCL1 binding is associated with neuronal developmental processes, whereas SG2M enriched binding is uniquely associated with mitotic and cell cycle processes, (although we do now see some axonal terms appear under these less stringent conditions). These two figures have now been added to Supplementary Figure 4.

© 2023. Published by The Company of Biologists under the terms of the Creative Commons Attribution License (https://creativecommons.org/licenses/by/4.0/).

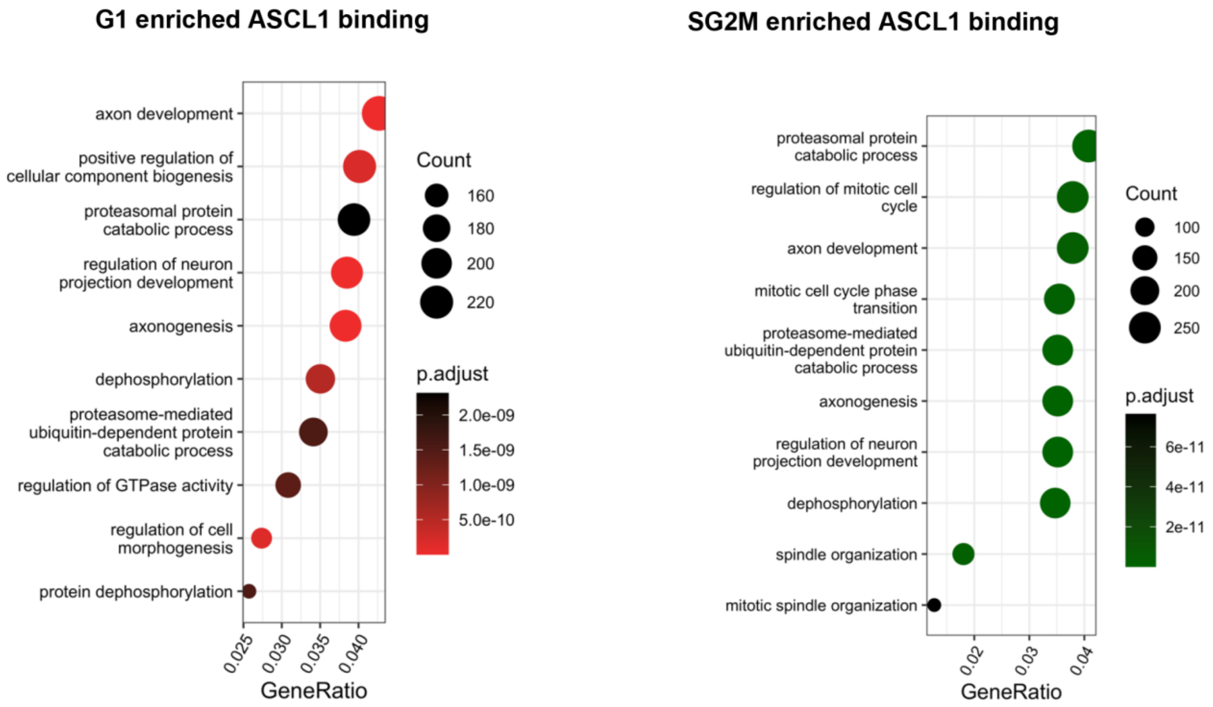

"The correlate the genes that decline with ASCL1 KO and the peaks from the ChIP-seq using GO terms, but would be very useful to determine how many of these genes are direct targets. This can bve done by showing the correlaiton between the RNAseq and the ChIP-seq on a gene-by-gene basis rather than using GO."

Thank you for this useful suggestion. To investigate any correlation between the ASCL1 ChIP- seq and ASCL1 KO RNA-seq, we quantified the log2 fold change in expression level (WT/KO) following ASCL1 KO for any gene that was associated with an ASCL1 binding site in asynchronous cycling cells. Plotting these fold changes as a histogram/density plot (left) reveals that these genes generally exhibited a positive fold change i.e. a decrease in expression level following ASCL1 KO (blue dotted line shows the mean log2 fold change for the ASCL1 bound genes, black dotted line is at 0). Looking specifically at the 1000 genes associated with the most significant ASCL1 ChIP-seq peaks confirms this (right), where more genes show large decreases in gene expression following KO, where the local polynomial regression (LOESS; locally estimated scatterplot smoothing, black line) is consistently higher than 1.

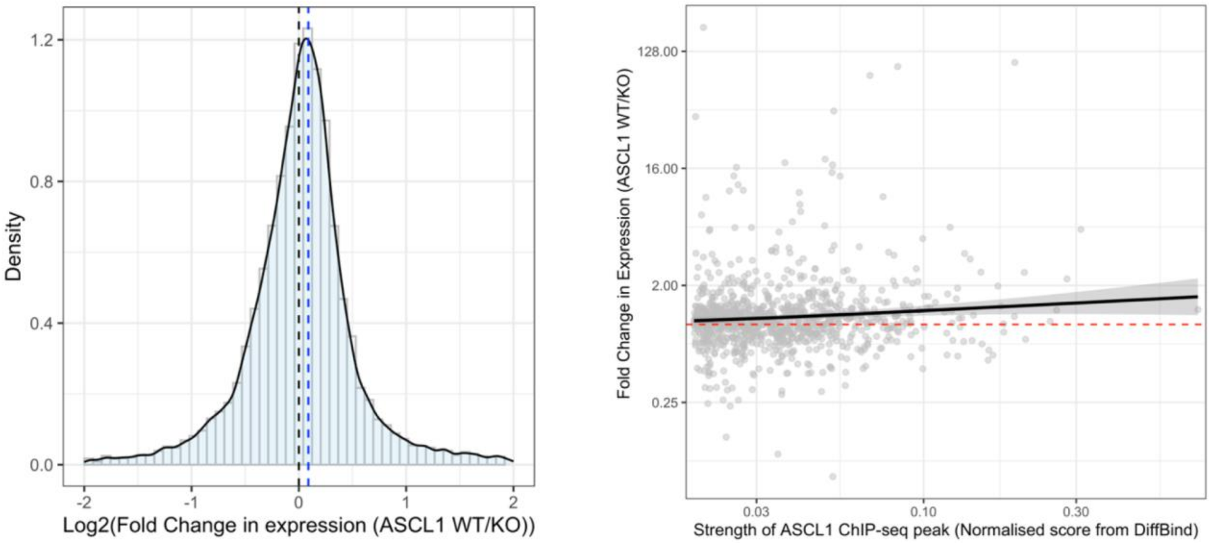

Left plot: Log2 fold change in expression level for all ASCL1 bound genes, where positive fold change indicates a reduction in expression level following ASCL1 knockout, and a negative fold

© 2023. Published by The Company of Biologists under the terms of the Creative Commons Attribution License (https://creativecommons.org/licenses/by/4.0/).

change indicates an increased expression following knockout. The mean value (blue dotted line), mode and median are all greater than 0 (black dotted line) indicating general reduction in expression level following ASCL1 knockout.
Right plot: 1000 genes associated with the strongest ASCL1 peaks (normalised peak score from DiffBind) were plotted against their fold change in expression following ASCL1 knockout. There is a large amount of variability, but the local polynomial regression (LOESS, black line) is consistently greater than 1 (red dotted line; no fold change).

We have now added the right figure to Supplementary Figure 2

Reviewer 2 also raised similar concerns:

“Other minor points:
In figure 2, it would be interesting to display the overlap between bound and regulated genes.”

As suggested, we looked at the overlap between genes bound by ASCL1 in DMSO treated, freely cycling cells and intersected them with genes that showed a significant change in expression level following ASCL1 KO. This reveals that the majority of bound genes are regulated by ASCL1. Put another way, the large majority of genes that exhibited differential expression following ASCL1 KO were bound by ASCL1 in WT cells:

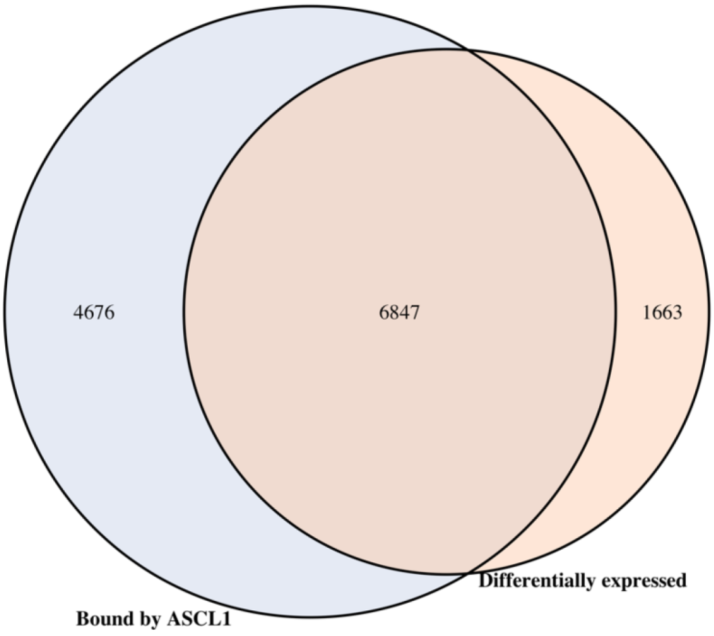

We have now added this Venn diagram to Figure 2.

“The lack of ASCL1 dependence of the G1 neuronal genes (Fig 5B) is interesting, but may be confounded by the possibility that these sites are driven equally well by a redundant proneural trnascription factor, like NEUROD1 or NEUROG. This possibility should be addressed by carrying out ChIP for these factors at select sites (G2M vs G1). Alternatively ChIP-seq for these factors would be ideal. Without these experiments the conclusion is not supported: "This indicates that ASCL1 is capable of binding to neuronal targets in G1 phase of the cell cycle in neuroblastoma cells but is not supporting their expression under cycling conditions.

The problem of redundant TFs is also an issue with the experiments to teat the effects of long G1 arrest.”

Thank you for raising this possibility, which prompted us to look at expression of other proneural proteins in these neuroblastoma cells. Consistent with the important role for ASCL1 in neuroblastoma previously reported in contrast to lack of reports about prominent roles for other proneural transcription factors, we quantified the expression levels of other proneural proteins in

© 2023. Published by The Company of Biologists under the terms of the Creative Commons Attribution License (https://creativecommons.org/licenses/by/4.0/).

parental SK-N-BE(2)-C cells and the ASCL1 KO clone. We found that the expression level of all other proneural factors was very low, especially when compared to ASCL1, and did not increase following ASCL1 KO, showing no signs of compensatory uplift. We therefore conclude that there is a very low likelihood of interference from these factors. Moreover, methodologies such as ChIP-seq for these other proneural proteins are unlikely to work given their extremely low expression levels. We now include these findings in Supplementary Figure 5.

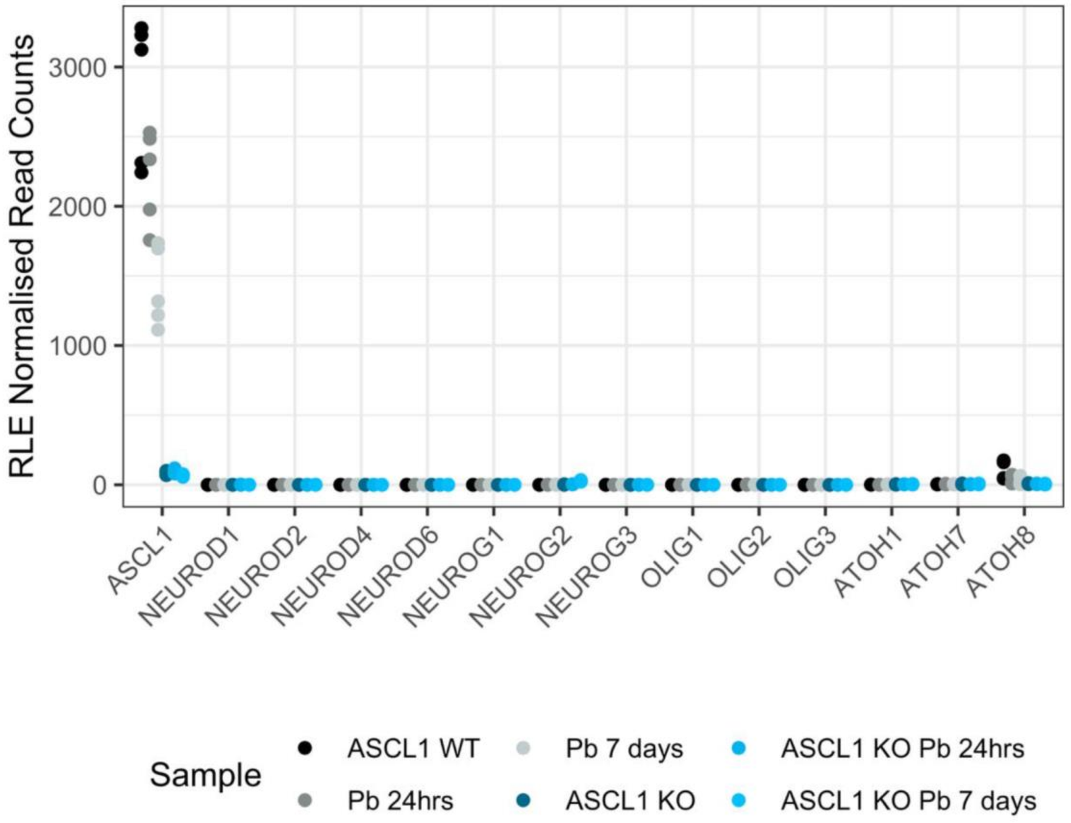

"The finding that G1 ASCL1 sites show less accessibility than G2M sites is interesting; is thre a reduction in ASCL1 ChIP-seq signal at these sites as well? Or is ASCL1 bound but not able to open the chromaitn at these sites?"

We have shown in Supplementary Figure 3 of the original manuscript that there is a reduced level of ASCL1 binding at G1 enriched sites compared to SG2M enriched sites when looking at asynchronous, freely cycling cells SK-N-BE(2)-C, and two other neuroblastoma cell lines.

To further investigate this, we performed this same analysis on the individual SK-N-BE(2)-C asynchronous replicates independently, which showed the same trend. These freely cycling cells comprise approximately 65% G0/G1 cells and 35% SG2M cells (Figure 3C). Despite more cells being in G1 in asynchronous freely cycling cells, the ASCL1 ChIP-seq signal is markedly reduced for sites which are preferentially bound by ASCL1 during G1 phase. Addressing the Reviewer's question, this indicates that the lower levels of accessibility at G1 enriched sites versus G2M enriched sites are a result of reduced binding of ASCL1 in G1.

© 2023. Published by The Company of Biologists under the terms of the Creative Commons Attribution License (https://creativecommons.org/licenses/by/4.0/).

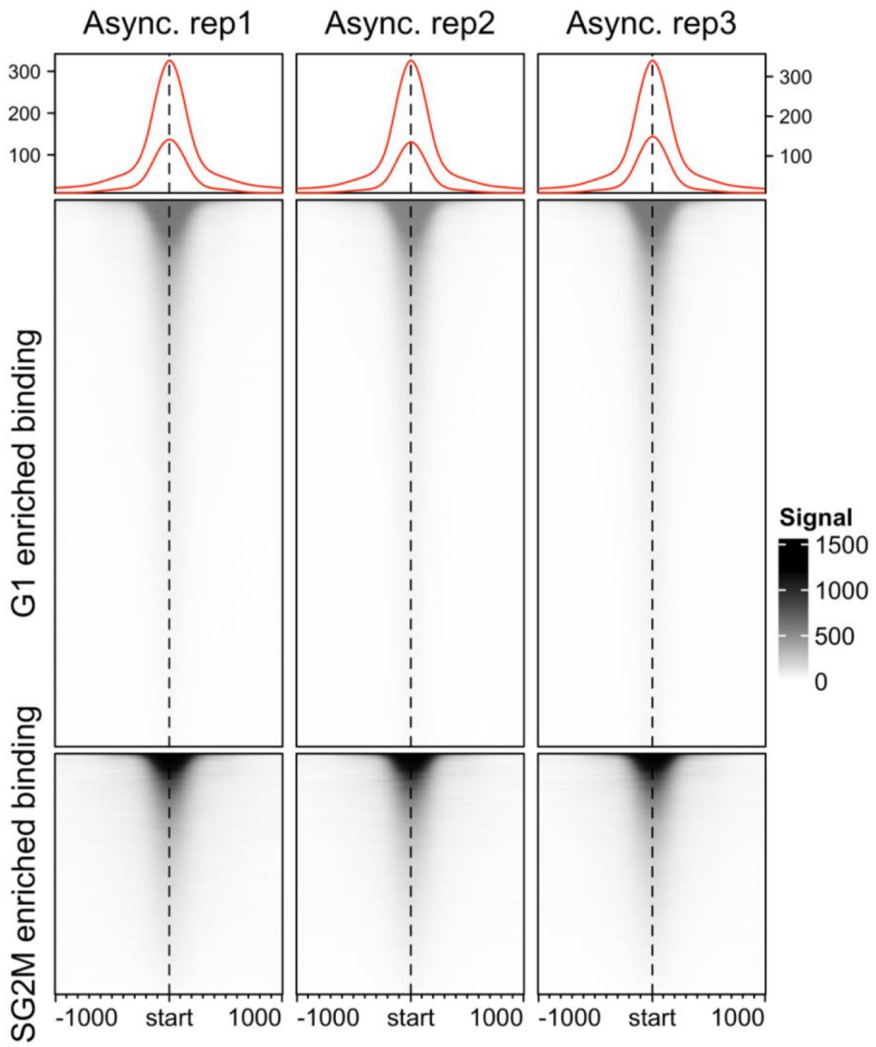

We hypothesised that reduced binding in G1 could be a result of lower ASCL1 protein concentrations. To address this, we performed ASCL1 antibody-based staining and hoechst based cell cycle analysis in SK-N-BE(2)-C cells, followed by flow cytometry. This enabled us to individually quantify ASCL1 protein levels in specific cell cycle subpopulations. The relative cell size changes across the cell cycle, so to account for this we plotted the relative changes in ASCL1 protein levels with the relative changes in cell size. This revealed that ASCL1 protein levels in G2M were significantly higher than expected if solely due to changes in cell size (and the levels in S phase were lower than expected for the cell size). In contrast, when we performed the same analysis for the housekeeping gene, TBP, we observed more consistent protein levels that scaled proportionately with cell size. This reveals a degree of cell cycle- dependent regulation of ASCL1 protein levels, which may account for differences in overall binding between the two phases, and indicate that reduced ASCL1 binding in G1 may be due to a lower amount of ASCL1 protein compared to the level in other cell cycle stages (normalised for cell size).

© 2023. Published by The Company of Biologists under the terms of the Creative Commons Attribution License (https://creativecommons.org/licenses/by/4.0/).

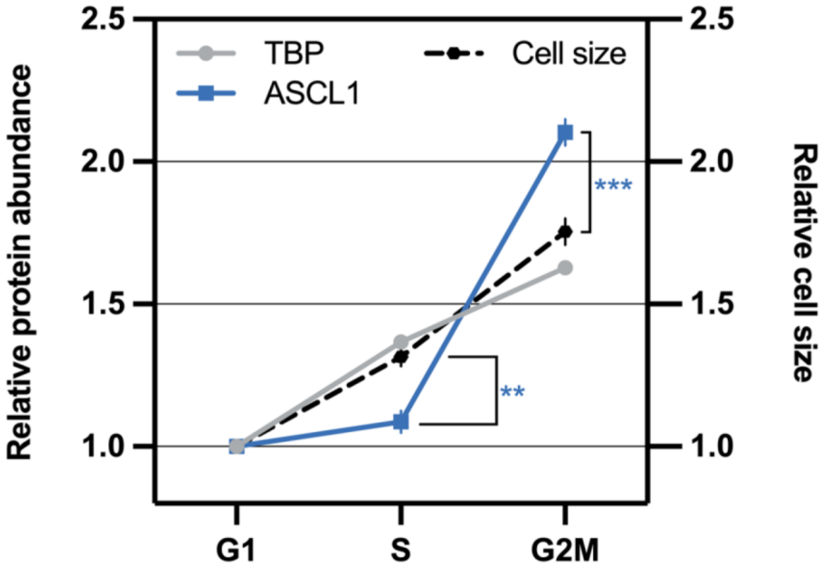

We have now moved the SK-N-BE(2)-C plot from original Supplementary Figure 3 to Figure 4, and added the results above to Figure 4.

"The reduction in accessible sites in the ASCL1 KO for the G2M sites is consistent with the effects on proliferation, but the effect is very modest. Would this effect be greater if the analysis of the ATAC-seq data were confined to sites with E-boxes? it would be useful to know what percentage of the accessible sites have an E-box and what percent of these sites are lost in the ASCL1 KO. This might show the importance of redundant proneural TFs."

We now undertake additional analysis to address this important point directly. Of the 14,460 peaks that exhibit enriched ASCL1 binding during SG2M, 9,228 contain a canonical ASCL1 E- box motif (NNVVCAGCTGBN, taken from HOMER motif analysis above), as determined by FIMO, MEME suite (q-value <0.05).

We quantified the ATAC-seq signal at these peaks containing high confidence ASCL1 E-box motifs before and after ASCL1 KO and found that this extra filtering step had no impact on the magnitude of the change in accessibility following ASCL1 KO. This suggests that ASCL1 knockout has an equal effect on the accessibility of bound sites regardless of the underlying motif, and indirectly indicates that even the peaks showing degenerate ASCL1 motifs show a reduction in accessibility following ASCL1 knockout. This latter set could include sites where ASCL1 binding is mediated or enhanced by a cofactor.

© 2023. Published by The Company of Biologists under the terms of the Creative Commons Attribution License (https://creativecommons.org/licenses/by/4.0/).

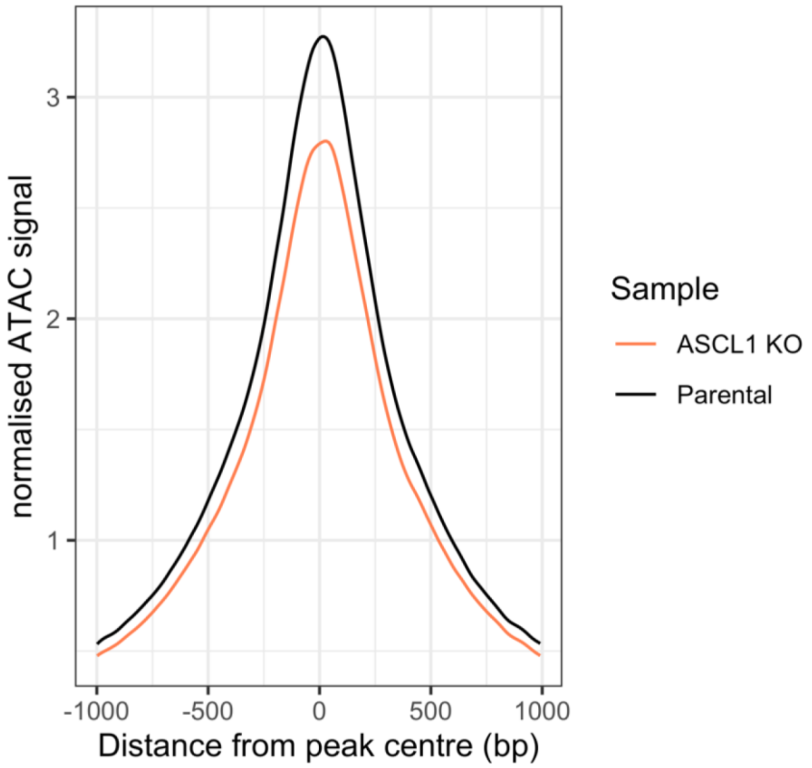

Reviewer 2:

"There is however, one important concern to be clarified before strong conclusions can be extracted from the data: are palbociclib-treated cells comparable to control cells? 7 days of G1 arrest could have led to differentiation of at least a fraction of the NSCs and therefore the increased expression of neuronal genes (and chromatin changes) could reflect a higher percentage of differentiated cells (or higher degree of differentiation) in that sample rather than increased expression of neuronal genes in NSCs. A characterization of the cultures after the 7- day treatment is therefore necessary before drawing any conclusions. This could be done through immunohistochemistry to assess the presence of differentiated cells and control for the continuous and homogeneous expression of stemness markers (some useful markers include Nestin, Sox2, DCX, Tubb3 or GFAP). The reversibility assay, as shown in Figure S2 would also be very informative for the 7-day time point."

For ASCL1 ChIP-seq experiments on cell cycle synchronized cells, palbociclib treatment was for a short duration of 24 hours, to ensure that the cells are only stalled in G1, and not differentiating. Control cells were treated with DMSO for the same duration, and the confluency was not more than 80% to ensure that they are healthy, cycling cells.

It was not experimentally possible to directly compare cells plated at the same density and then grown with or without PB for 7 days as extreme overgrowth and extensive cell death (rather that cell cycle arrest and differentiation) occurred in the cells without PB. When we performed 7 day palbociclib treatments, we plated control cells at half the density of treated cells so that by the 7 day time point, they were not overly confluent and were still cycling, allowing us to collect control cells for the RNA-seq analysis comparison. The morphology of the 7 day PB-treated cells were markedly different from control cells, showing extended neurites and overall lower confluency due to cell cycle exit and differentiation (see below).

The morphological effects of PB treatment on neuroblastoma cells was covered in some detail in a previous publication, Ferguson et al, 2023, Dev Cell, 58:1967-1982 . In this previous study we have extensively characterised the morphology of SK-N-BE(2)-C cells plated under very similar conditions to those used here, DMSO treated (again plated on day 0 at a lower density that PB treated to limit control cell death) versus palbociclib treated, below,). These cells were stained for Tubb3 as suggested by the Reviewer. We saw extensive cell cycle inhibition morphological differentiation

© 2023. Published by The Company of Biologists under the terms of the Creative Commons Attribution License (https://creativecommons.org/licenses/by/4.0/).

with PB accompanied by upregulation of Tubb3 and neurite extension. In contrast we saw very little Tubb3 upregulation or morphological change in the DMSO control cells, and cells maintain a largely uniform typical neuroblast morphology. We now describe this previous work that directly addresses the point raised more fully in the results and discussion of this manuscript.

NOTE: Figure provided for reviewer has been removed. It showed Figure 1E and 1H from Ferguson KM, Gillen SL, Chaytor L, Poon E, Marcos D, Gomez RL, Woods LM, Mykhaylechko L, Elfari L, Martins da Costa B, Jamin Y, Carroll JS, Chesler L, Ali FR, Philpott A. Palbociclib releases the latent differentiation capacity of neuroblastoma cells. Dev Cell. 2023 Oct 9;58(19):1967-1982.e8. doi: 10.1016/j.devcel.2023.08.028. Epub 2023 Sep 20. PMID: 37734383. **We have removed unpublished data that had been provided for the referees in confidence.**

Figure from Ferguson et al., 2023.

To further address the point raised by Reviewer 2, we undertook more interrogation of our RNAseq data to confirm that 7 days of palbociclib treatment is inducing differentiation compared to the control cells. Taking suggestions from the Reviewer, we quantified the expression of several markers of stemness and neuronal differentiation from the RNA-seq data comparing treated and untreated cells. Indeed, the stemness markers SOX2, MYCN and HES1 all decrease following treatment, while the expression of key early neuronal genes (DCX, MAP2) increases.

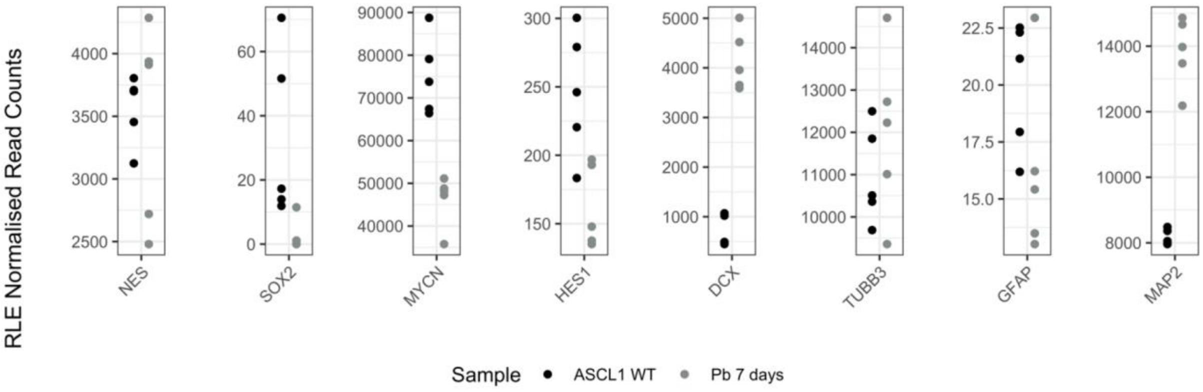

We have now added this plot to Supplementary Figure 4.

"Other minor points:
In figure 2, it would be interesting to display the overlap between bound and regulated genes."
As suggested, we looked at the overlap between genes bound by ASCL1 in DMSO treated, freely cycling cells and intersected them with genes that showed a significant change in expression level following ASCL1 KO. This reveals that the majority of bound genes are regulated by ASCL1. Put another way, the large majority of genes that exhibited differential expression following ASCL1 KO were bound by ASCL1 in WT cells:

© 2023. Published by The Company of Biologists under the terms of the Creative Commons Attribution License (https://creativecommons.org/licenses/by/4.0/).

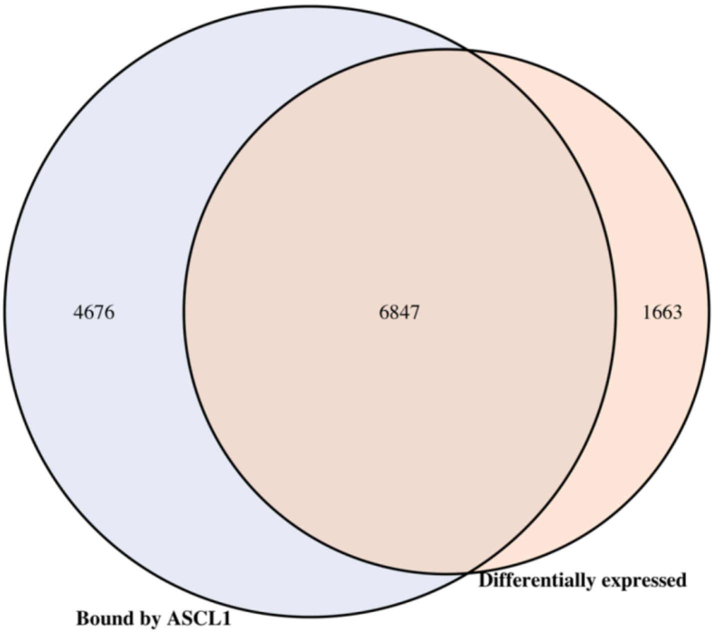

We have now added this Venn diagram to Figure 2.

"Please clarify where does the number of 47,294 non-commonly regulated genes between G1 and S/G2/M come from. From the data in figure 3D the number should be roughly 30k."

Thank you for raising this. We agree that this is not clear and have changed the text and figure legend to better explain it. Prior to DiffBind analysis, the consensus peak sets for palbociclib-treated cells and thymidine-treated cells are shown in figure 3D. A consensus peak is one that appears in two out of the three replicates for that condition. DiffBind is then run using these consensus peak sets, which takes the magnitude of the peaks into account, identifying 47,294 differentially bound regions.

"In figure 3F/G, it would be very informative to show also examples of cell cycle independent genes."

Recognising this was a minor point, we would suggest that this is largely a control for cell cycle-dependent expression that is extensively analysed in the rest of the paper. Unfortunately we do not have any remaining ChIP'ed DNA with which to show control regions. The samples were generated from approx. 1 million FACS sorted cells and so all ChIP'ed DNA was used for the qPCR reactions shown.

"In graph 4B, please unify the way the legend is displayed (location of "count" and "p.adjust")."

Corrected in the figure.

© 2023. Published by The Company of Biologists under the terms of the Creative Commons Attribution License (https://creativecommons.org/licenses/by/4.0/).

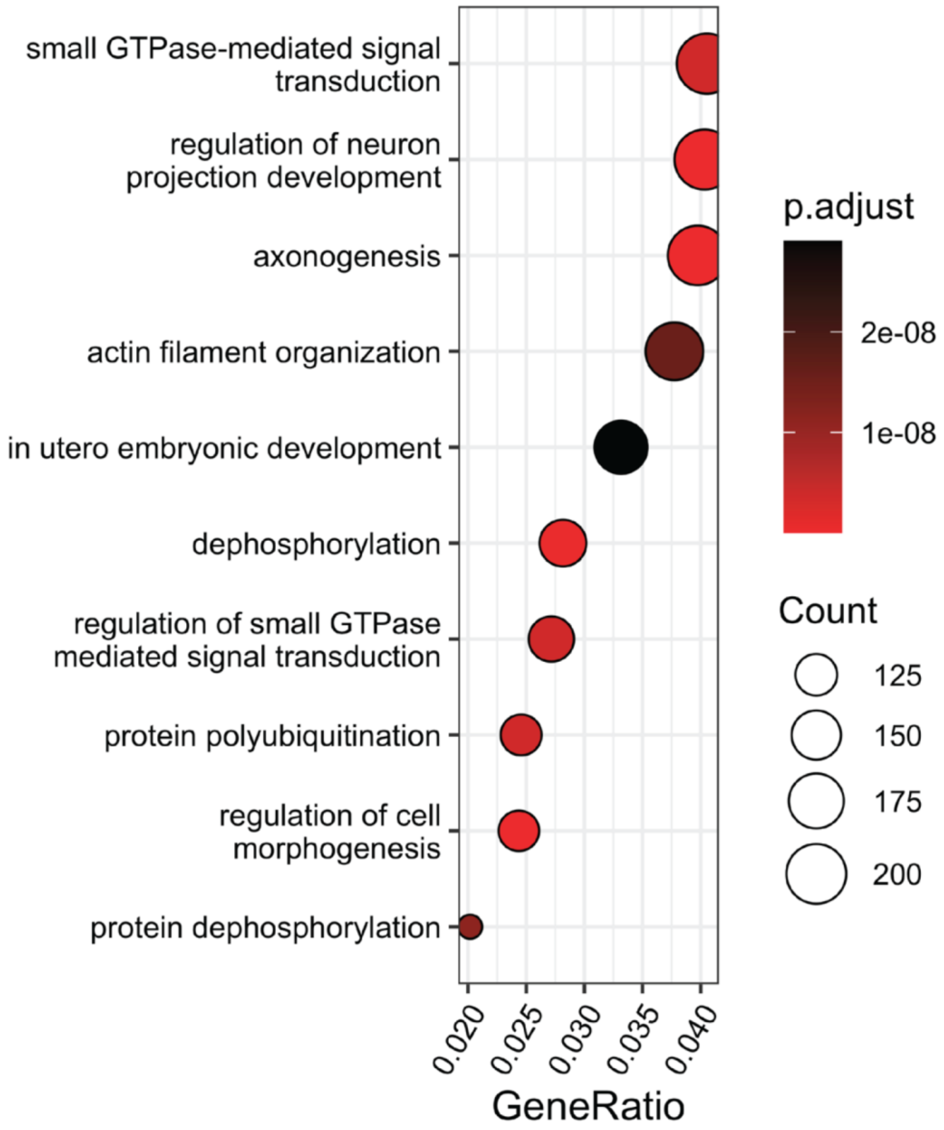

"In figure 5A, could it be that the expression levels of neuronal genes are too low in control cells, so that it is difficult to see a difference in the cKO cells? Even if not significant, would be good to show the p value."

It is certainly possible that expression of neuronal genes is low in the WT cells and that this is why ASCL1 KO has no significant effect, but it still raises the question of how ASCL1 can bind and not drive the expression of these genes in this context. We would expect the statistical test to identify significant differences regardless of the expression level.

Since there are multiple t tests performed in each of the right figure panels, we used the Bonferroni's Correction for multiple testing which is equal to the p-value divided by the number of statistical tests performed (i.e. 0.05/7 = 0.0071). Thus, any test with an adjusted p-value higher than 0.0071 is considered non-statically significant.

© 2023. Published by The Company of Biologists under the terms of the Creative Commons Attribution License (https://creativecommons.org/licenses/by/4.0/).

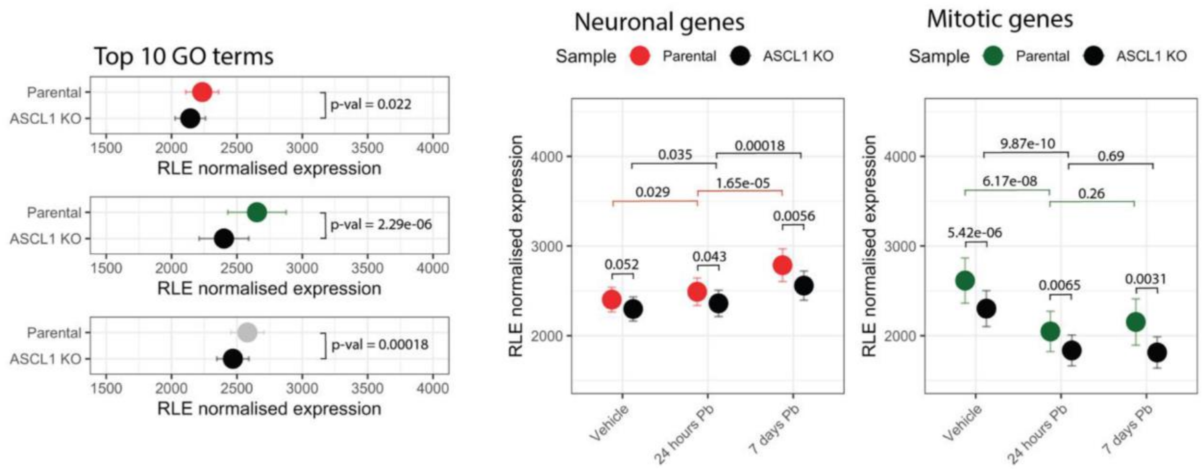

We have now updated the figure to show the p-values, and will modify the figure legend to explain the multiple testing correction. Additional information has also been added to the methods section.

“And simply a style point: I found the color scheme for significance in the graphs confusing, as dark colors signify less significance and white/clear shades high significance.”

For all other GO analyses figures, we have used a colour to represent high significance and black to represent lower significance, and it is for this reason that the GO analyses in Figures 1 and 2 use black to represent low significance. For consistency we feel it is best to keep it the same throughout the paper.

Original submission

First decision letter

MS ID#: dev.204816

MS TITLE: Distinct proliferative and neuronal programmes of chromatin binding and gene activation by ASCL1 are cell cycle stage-specific

AUTHORS: William F. Beckman; Lydia Parkinson; Lewis Chaytor; Anna Philpott

Dear Anna,

Thank you for sending your manuscript to Development through Review Commons.

I have now received all the referees reports on the above manuscript, and have reached a decision. The referees' comments are appended below, or you can access them online: please go to .

The overall evaluation is positive and we would like to publish a revised manuscript in Development, provided that you satisfactorily address the remaining request of referee 1. Please attend to this comment in your revised manuscript and in your point-by-point response. If you do not agree with their suggestion, explain clearly why this is so.

Reviewer 1: COMMENTS ON TEXT

The authors have adequately replied to my comments and suggestions. They also confirmed that 7 days of treatment do induce neuronal differentiation and therefore the cells at the end of the treatment are not stem cells anymore. A statement should be added after the conclusions of figure

© 2023. Published by The Company of Biologists under the terms of the Creative Commons Attribution License (https://creativecommons.org/licenses/by/4.0/).

5 and/or the discussion opening the possibility that the differences in the expression of neuronal Ascl1 targets are not (or not only) due to direct binding of Ascl1 to those regions but also to differences in the percentage of neurons present in the culture (which will be presumably lower in the Ascl1KO cells).

Reviewer 2: COMMENTS ON TEXT

The authors have done a really nice job of addressing prior concerns with additional analyses and data. I have no additional concerns

---

First revision

Author response to reviewers' comments

Response to Reviewers:

Apologies for the slight delay in returning our manuscript due to illness and travel. We thank the reviewers for the further thoughts and are delighted that the paper is acceptable for publication in Development with very minor changes. In response to Reviewer 1 we have added the requested statement associated with Figure 5 (on Page 9, “Consistent with these previous findings, we observe a strong downregulation of stemness markers SOX2, MYCN and HES1, and a strong upregulation of neuronal genes DCX and MAP2 following 7 days of palbociclib treatment (Fig. S5A), which may represent the increased fraction of neurons versus progenitor cells in the culture”. We hope the paper is now ready for formal acceptance and we look forward to seeing it in print.

Second decision letter

MS ID#: dev.204816R1

MS TITLE: Distinct proliferative and neuronal programmes of chromatin binding and gene activation by ASCL1 are cell cycle stage-specific

AUTHORS: William F. Beckman; Lydia Parkinson; Lewis Chaytor; Anna Philpott
ARTICLE TYPE: Research Article

Dear Anna,

I am delighted to tell you that your manuscript has been accepted for publication in Development, pending our standard publication integrity checks.

© 2023. Published by The Company of Biologists under the terms of the Creative Commons Attribution License (https://creativecommons.org/licenses/by/4.0/).
